# An empirical evaluation of approximate and exact regression-based causal mediation approaches for a binary outcome and a continuous or a binary mediator for case-control study designs

**DOI:** 10.1186/s12874-024-02156-y

**Published:** 2024-03-20

**Authors:** Miguel Caubet, Kevin L’Espérance, Anita Koushik, Geneviève Lefebvre

**Affiliations:** 1 Department of Mathematics, Université du Québec à Montréal, Montreal, Canada; 2 Department of Social and Preventive Medicine, Université de Montréal, Montreal, Canada; 3 Université de Montréal Hospital Research Centre (CRCHUM), Montreal, Canada; 4 St. Mary’s Research Centre, Montreal, Canada; 5 Department of Oncology, Faculty of Medicine and Health Sciences, McGill University, Montreal, Canada

**Keywords:** Mediation analysis, Counterfactuals, Binary outcomes, Natural effects, Case-control study, Rare outcome assumption, Ovarian cancer, Oral contraceptives

## Abstract

**Background:**

In the causal mediation analysis framework, several parametric regression-based approaches have been introduced in past years for decomposing the total effect of an exposure on a binary outcome into a direct effect and an indirect effect through a target mediator. In this context, a well-known strategy involves specifying a logistic model for the outcome and invoking the rare outcome assumption (ROA) to simplify estimation. Recently, exact estimators for natural direct and indirect effects have been introduced to circumvent the challenges prompted by the ROA. As for the approximate approaches relying on the ROA, these exact approaches cannot be used *as is* on case-control data where the sampling mechanism depends on the outcome.

**Methods:**

Considering a continuous or a binary mediator, we empirically compare the approximate and exact approaches using simulated data under various case-control scenarios. An illustration of these approaches on case-control data is provided, where the natural mediation effects of long-term use of oral contraceptives on ovarian cancer, with lifetime number of ovulatory cycles as the mediator, are estimated.

**Results:**

In the simulations, we found few differences between the performances of the approximate and exact approaches when the outcome was rare, both marginally and conditionally on variables. However, the performance of the approximate approaches degraded as the prevalence of the outcome increased in at least one stratum of variables. Differences in behavior were also observed among the approximate approaches. In the data analysis, all studied approaches were in agreement with respect to the natural direct and indirect effects estimates.

**Conclusions:**

In the case where a violation of the ROA applies or is expected, approximate mediation approaches should be avoided or used with caution, and exact estimators favored.

**Supplementary Information:**

The online version contains supplementary material available at 10.1186/s12874-024-02156-y.

## Introduction

Mediation analysis aims at decomposing the effect of an exposure on an outcome into a direct effect and an indirect effect through a target mediator, thereby allowing for a better understanding of the mechanisms by which the exposure affects the outcome [1]. In causal mediation analysis, the total effect decomposition is accomplished via *natural* mediation effects [2, 3]. When the outcome is binary, standard regression-based approaches for the estimation of natural effects use a logistic model for the outcome and either a linear or a logistic model for the continuous or binary mediator, respectively [4]. Well-known approaches for performing causal mediation analysis with a binary outcome were developed using the rare outcome assumption (ROA), yielding simplified inference [5, 6]. To address the approximate nature of these approaches, there has recently been an interest in developing so-called *exact* regression-based approaches for natural effects estimation, where the exact estimators circumvent the ROA and are applicable independently of the rareness or commonness of the outcome [7–11].

In Samoilenko, Blais and Lefebvre [7] and Samoilenko and Lefebvre [8, 9], the authors found that the exact estimators yielded more accurate estimates of natural effects than the approximate ones in simulation scenarios where the outcome was common, but also when the outcome was rare marginally but not conditionally. It is relevant to mention that these studies implemented and compared approaches on data which allowed estimation of the outcome and mediator models’ parameters consistently using standard fitting procedures, that is, on data arising from cohort or population-based designs. As the case-control design is indicated when the frequency of the outcome is small or smaller than that of the exposure [12], it is pertinent to investigate whether the gain from using an exact mediation approach that does not rely on the ROA is preserved when implemented on case-control data. As detailed in the sequel, implementation of approximate and exact approaches on case-control data requires more attention than when implemented on cohort data since some parameters of the outcome and mediator models might not be estimated consistently using standard fitting procedures (e.g., [5, 6, 13, 14]). Causal mediation analysis with case-control data has been discussed in key articles such as [5, 6, 13], and a number of studies have been performed to shed further light on this topic [14–17]. However, as the latter articles do not specifically address the comparison of approximate versus exact approaches, we believe it worthwhile to investigate this issue.

As alluded to previously, approximate mediation analysis approaches relying on the ROA should not be used *as is* on case-control data where the sampling mechanism depends on the outcome. This well-known fact occurs as a result of the selection of individuals based on their outcome status, which can yield biased estimators of the regression coefficients of the mediator model. Such a bias notably happens when there are arrows pointing from the exposure to the outcome and from the mediator to the outcome in a causal mediation diagram. For these approaches, Valeri and VanderWeele [6] have proposed to account for the case-control design by fitting the mediator model on the controls only. Alternatively, VanderWeele and Vansteelandt [5] and Valeri and VanderWeele [6] have suggested using inverse probability weighting (IPW) for fitting this model, but implementing IPW requires knowledge of the frequency of the outcome, the latter interpreted either as a prevalence or an incidence. Similar issues regarding the estimation of the mediator model prevail for the exact approaches. However, there is an additional difficulty for these, since, unlike for the approximate approaches [5], the case-control design also needs to be accounted for when estimating the outcome logistic model. This occurs because the intercept coefficient of this model is involved in the definition of the exact natural direct and indirect effects estimands [8, 9].

The objective of this work is to empirically compare the performance of approximate versus exact approaches for the estimation of natural mediation effects odds ratios (ORs) in the context of case-control study designs where the outcome is binary and the mediator is either continuous or binary. In our comparisons, we focus on the regression-based approximate approaches available in the R package CMAverse [18], where the estimation of the mediator model is done according to either of the two aforementioned strategies. Similarly, to account for the case-control design in the exact approach, we use the IPW strategy implemented in the R package ExactMed [19], where the weighting is applied to both the mediator and outcome models. We also consider the unified likelihood approach of Satten et al. [17], which accounts for the case-control design using a joint prospective likelihood for the outcome and mediator. This approach does not require knowledge of the outcome prevalence (incidence) but nevertheless relies on the ROA.

The article is structured as follows. In Methods section, we present the definitions of mediation effects and associated models, and provide details on the approaches compared. In this section, we also describe the simulation study performed and report on the results in Results section. In Real data analysis section, we apply the approaches compared on case-control data from the PRevention of OVArian Cancer in Quebec (PROVAQ) study [20]. These analyses are performed to evaluate the direct and indirect effects of long-term use of oral contraceptives on ovarian cancer using the lifetime number of ovulatory cycles as the potential mediator.

## Methods

### Definitions, models and approaches

We first define the nested counterfactual outcome $Y(a,M(a^*))$ which is the outcome that would be realized if the exposure were set to *a* and the mediator were set to the value it would have taken if the exposure had been set to $a^*$. The conditional natural direct effect (NDE) and natural indirect effect (NIE) ORs corresponding to a change in the exposure level from $A = a^*$ to $A = a$ are defined as follows:

1 $$OR^{NDE}_{a, a^* }(\boldsymbol{c}) = \frac{\frac{\mathrm{P}(Y(a,\, M(a^*)) = 1 |\boldsymbol{C} = \boldsymbol{c})}{1 - \mathrm{P}(Y(a,\, M(a^*)) = 1 |\boldsymbol{C} = \boldsymbol{c})}}{ \frac{\mathrm{P}(Y(a^*,\, M(a^*)) = 1 |\boldsymbol{C} = \boldsymbol{c})}{1 - \mathrm{P}(Y(a^*,\, M(a^*)) = 1 |\boldsymbol{C} = \boldsymbol{c})}},$$

2 $$OR^{NIE}_{a, a^*}(\boldsymbol{c}) = \frac{\frac{\mathrm{P}(Y(a,\, M(a)) = 1 |\boldsymbol{C} = \boldsymbol{c})}{1 - \mathrm{P}(Y(a,\, M(a)) = 1 |\boldsymbol{C} = \boldsymbol{c})}}{ \frac{\mathrm{P}(Y(a,\, M(a^*)) = 1 |\boldsymbol{C} = \boldsymbol{c})}{1 - \mathrm{P}(Y(a,\, M(a^*)) = 1 |\boldsymbol{C} = \boldsymbol{c})}}.$$

The total effect (TE) OR is defined as the product of the NDE and NIE ORs:

$$\begin{aligned} OR^{TE}_{a, a^* } (\varvec{c})= OR^{NDE}_{a, a^*}(\varvec{c}) \times OR^{NIE}_{a, a^*}(\varvec{c}). \end{aligned}$$

Identification of natural direct and indirect effects is accomplished via the mediation formula [21], which is established using consistency, positivity and conditional independence assumptions [22, 23]. Mediation formulas corresponding to a binary outcome and a continuous or binary mediator, respectively, are:

3 $$\begin{aligned}\mathrm P(Y(a,M(a^\ast))=1\vert\,\boldsymbol C=\boldsymbol c)&=\int\mathrm P(Y=1\vert\,A=a,M=m,\boldsymbol C=\boldsymbol c)\\ &\quad \times f(M=m\vert\,A=a^\ast,\boldsymbol C=\boldsymbol c)\,dm,\end{aligned}$$

4 $$\begin{aligned} \mathrm P(Y(a,M(a^\ast))=1\vert\,\boldsymbol C=\boldsymbol c)&=\sum_m\mathrm P(Y=1\vert\,A=a,M=m,\boldsymbol C=\boldsymbol c)\\ &\quad \times P(M=m\vert\,A=a^\ast,\boldsymbol C=\boldsymbol c),\end{aligned}$$

where $\varvec{C}$ is a set of covariates sufficient to achieve ignorability for the $A-Y$, $A-M$, and $M-Y$ relationships [4].

Throughout, we assume the following logistic regression model for *Y* for consideration in mediation formulas (3-4):

5 $$\text{logit}(\mathrm{P}(Y = 1 | A =a, M = m, \boldsymbol{C} = \boldsymbol{c})) = \theta_0 + \theta_1 a + \theta_2 m + \theta_3 a m + \boldsymbol{\theta}_4' \boldsymbol{c}.$$

Moreover, we use either of the following linear or logistic models, respectively:

6 $$\begin{aligned}&\mathbb {E}(M | A = a, \boldsymbol{C} = \boldsymbol{c}) = \beta _0 + \beta _1 a + \boldsymbol{\beta }^{\prime}_{2} \boldsymbol{c} , \end{aligned}$$

7 $$\text{logit}(\mathrm{P}(M = 1 | A = a, \boldsymbol{C} = \boldsymbol{c})) = \beta_0 + \beta_1 a + \boldsymbol{\beta}_2^{\prime} \boldsymbol{c}.$$

In the case of a continuous *M*, a Gaussian distribution is assumed for the mediator in formula (3), that is $M =\mathbb {E}(M | A, \varvec{C}) + \epsilon$ with $\epsilon \sim N(0, \sigma ^2)$ and *f* is the corresponding density, while model (7) is used in formula (4).

#### Standard approximate approaches

The regression-based approaches for a binary outcome and a continuous or binary mediator proposed by VanderWeele and Vansteelandt [5] and Valeri and VanderWeele [6] for the estimation of natural direct and indirect effects on the OR scale ($OR^{NDE}$ and $OR^{NIE}$) rely on models (5) and either (6) or (7); these approaches also invoke the ROA for providing approximate closed-form expressions for the natural effects. For a binary exposure coded 0/1, the expressions pertaining to a continuous mediator are:

8 $$\begin{aligned} & OR^{NDE}_{1,0; \,app}(\boldsymbol{c}) = \exp (\theta _{1}+\theta _{3}(\beta _{0}+\boldsymbol{\beta }_2^{\prime} \boldsymbol{c}+\theta _{2}\sigma^{2}) + 0.5 \theta _{3}^{2} \sigma^{2} ),\end{aligned}$$

9 $$\begin{aligned} & OR^{NIE}_{1,0; \,app}(\boldsymbol{c}) = \exp (\beta _{1} (\theta _{2}+ \theta _{3})), \end{aligned}$$

while, for a binary mediator, these are:

10 $$\begin{aligned} OR^{NDE}_{1,0; \,app}(\varvec{c}) = \frac{\exp (\theta _1)(1+\exp (\theta _2+\theta _3+\beta _0+\varvec{\beta }_2^{\prime} \varvec{c}))}{1+\exp (\theta _2+\beta _0+\varvec{\beta }_2^{\prime} \varvec{c})}, \end{aligned}$$

11 $$\begin{aligned} OR^{NIE}_{1,0; \,app}(\boldsymbol{c}) &= \frac{(1+\exp(\beta_0+\boldsymbol{\beta}_2' \boldsymbol{c}))}{(1+\exp(\beta_0+\beta_1+\boldsymbol{\beta}_2' \boldsymbol{c}))} \\ &\quad \times \frac{(1+\exp(\theta_2+\theta_3+\beta_0+\beta_1+\boldsymbol{\beta}_2' \boldsymbol{c}))}{(1+\exp(\theta_2+\theta_3+\beta_0+\boldsymbol{\beta}_2' \boldsymbol{c}))}.\end{aligned}$$

The corresponding estimators for the natural effects ORs are obtained by substituting the parameters in (8-9) or (10-11) by corresponding estimators obtained according to the type of mediator.

Implementation of these approximate approaches can be done using the R package CMAverse with the cmest function and option rb. In the context of a case-control study, the first strategy, which consists of fitting the mediator model among the controls only, is implemented by setting the options casecontrol and yrare to TRUE. The theoretical basis for this strategy uses the argument that when the outcome is rare, one could assume that

12 $$\begin{aligned} \mathbb {E}(M = m |Y = 0, A =a, \boldsymbol{C}= \boldsymbol{c})\approx & \, \mathbb {E}(M = m |A =a, \boldsymbol{C}= \boldsymbol{c})\nonumber \\= & \, \beta _0 + \beta _1 a + \boldsymbol{\beta }'_2 \boldsymbol{c}, \end{aligned}$$

13 $$\begin{aligned} &\text{logit}(\text{P}({M} = 1 | {Y} = 0, {A} = {a}, \varvec{C}= \varvec{c}))\approx\\ & \text{logit}(\text{P}(M = 1 | {A} = {a}, \varvec{C}= \varvec{c})) = \beta _0 + \beta _1 {a} + \varvec{\beta }'_2 \varvec{c}. \end{aligned}$$

While the above equivalences are deemed valid when assuming the ROA, exclusion of cases when fitting the mediator model leads to a loss of efficiency for the estimators of the regression parameters of the mediator model. This is the rationale invoked by Satten et al. [17] for introducing their unified approach. More subtly, we conjecture, in light of Samoilenko and Lefebvre [8, 9], that equivalences (12) and (13) may not hold if one relies on the marginal prevalence (incidence) of the outcome only to establish the ROA. This is explored via simulation in the present work. The second strategy, based on IPW, is implemented in CMAverse by setting the option casecontrol to TRUE and specifying a value for the parameter yprevalence. Concretely, for this second strategy, each case is weighted by $w=\pi /p$ and each control by $w=(1-\pi )/(1-p)$, where *p* is the proportion of cases in the sample and $\pi$ is the outcome’s prevalence or incidence ($\equiv$ yprevalence) in the population. In general, there is an overrepresentation of cases in the sample as compared to the population (that is, $p> \pi$), and therefore each case (control) gets down weighted (up weighted) appropriately. These design-based weights are then used in a weighted regression using all the sample. A drawback of this second strategy, as opposed to the first one, is that it requires knowledge of the prevalence (incidence) of the outcome in the population.

#### Exact approaches

The exact regression-based approaches for a binary outcome and a continuous or binary mediator proposed by Samoilenko and Lefebvre [8, 9] estimate natural direct and indirect effects also from models (5) and either (6) or (7). However, these approaches do not invoke the ROA, at the cost of more complex estimators for the NDE and NIE.

Under these approaches, the model-based nested counterfactual outcome probabilities are not algebraically simplified, which yields

14 $$\begin{aligned} & \textrm{P}(\mathit{Y}(\mathit{a, M}(\mathit{a}^*)) = 1| \boldsymbol{C} = \boldsymbol{c}) = \nonumber \\ &\frac{1}{\sqrt{2\pi \sigma ^2}} \int _{-\infty }^\infty \text {expit}(\theta _0 + \theta _1 a + \theta _2 m + \theta _3 a m + \boldsymbol{\theta }^{\prime}_4 \boldsymbol{c}) \nonumber \\ &\qquad \qquad \qquad \qquad \qquad \times \exp \big(-\frac{(m - (\beta _0 + \beta _1 a^* + \boldsymbol{\beta }^{\prime}_2\boldsymbol{c}))^2}{2 \sigma ^2}\big) \,\mathit{d m} \end{aligned}$$

for a continuous (Gaussian) mediator, and

15 $$\begin{aligned}\textrm{P}(\mathit{Y(a, M}(\mathit{a}^*)) &= 1 |\boldsymbol{C} = \boldsymbol{c}) \nonumber \\ &= \text {expit}(\theta _0 + \theta _1 a + \theta _2 + \theta _3 a +\boldsymbol{\theta }_4^{\prime} \boldsymbol{c}) \text {expit}(\beta _0 + \beta _1 a^{*} + \boldsymbol{\beta }_2^{\prime} \boldsymbol{c}) \nonumber \\ & \quad + \text {expit}(\theta _0 + \theta _1 a + \boldsymbol{\theta }_4^{\prime} \boldsymbol{c}) (1 - \text {expit}(\beta _0 + \beta _1 a^{*} + \boldsymbol{\beta }_2^{\prime} \boldsymbol{c})) \end{aligned}$$

for a binary mediator, where $\text {expit}(\alpha ) = \frac{\exp (\alpha )}{1 + \exp (\alpha )}$. It should be noted that integral (14) does not allow for a closed-form formula [9], unlike the corresponding integral in the approximate approach.

Estimators for these probabilities are defined by first substituting the parameters in (14) or (15) by corresponding estimators, and for a continuous mediator, resorting to numerical integration for computing the integral in (14). The estimators for the probabilities are then plugged-back in (1) and (2) to provide estimators of natural effects on the OR scale.

Samoilenko and Lefebvre’s exact approaches are implemented in the R package ExactMed, where the functions exactmed_c and exactmed are available for estimating $OR^{NDE}$ and $OR^{NIE}$ according to the type of mediator (continuous or binary). The package allows for using an IPW strategy accounting for case-control data, also via the use of option yprevalence. The same weights as for the approximate approaches are used for fitting the mediator model. These weights are also used when fitting the outcome model to achieve consistent estimation for the intercept coefficient $\theta _0$ involved in (14-15). We refer readers to Additional file 1 for information on variance estimation and confidence intervals (CIs) for these approaches.

#### Unified likelihood approach

Satten et al. [17] recently introduced a joint likelihood approach for estimating natural effects ORs based on standard case-control data, assuming a binary outcome and either a continuous or a binary mediator. This so-called unified likelihood approach accounts for the case-control design while incorporating all case information in the likelihood and eliminating the need for a user-specified outcome prevalence (incidence) value. More precisely, they considered the joint likelihood $L_p = \prod _i \textrm{P}(\mathit{Y} = \mathit{y}_{\mathit{i}}, \mathit{M} = \mathit{m}_{\mathit{i}} |\mathit{A} = \mathit{a}_{\mathit{i}}, \boldsymbol{C}= \boldsymbol{c}_i)$, where *i* indexes individuals, which can be factored into

16 $$\begin{aligned} L_p = \prod _i \textrm{P}(\mathit{M} = \mathit{m}_{\mathit{i}}| \mathit{Y} = \mathit{y}_{\mathit{i}}, \mathit{A} =\mathit{a}_{\mathit{i}}, \boldsymbol{C}= \boldsymbol{c}_{\mathit{i}}) \textrm{P}(\mathit{Y} = \mathit{y}_{\mathit{i}}| \mathit{A} = \mathit{a}_{\mathit{i}}, \boldsymbol{C}= \boldsymbol{c}_{\mathit{i}}). \end{aligned}$$

As in the Valeri and VanderWeele’s [6] “controls only" strategy described previously, Satten et al. [17] proposed to model $\textrm{P}(\mathit{M}= \mathit{m} | \mathit{Y} =0,\mathit{A} =\mathit{a}, \boldsymbol{C}= \boldsymbol{c})$ invoking the ROA. For a binary mediator, their approach thus assumes that $\text {logit}(\textrm{P}(\mathit{M} = 1 | \mathit{Y} = 0, \mathit{A} = \mathit{a}, \boldsymbol{C}= \boldsymbol{c})) \approx \beta _0 + \beta _1 \mathit{a} + \boldsymbol{\beta }'_2 \boldsymbol{c}$. For a Gaussian mediator, a normal density function with mean $\mathbb {E}(M = m |Y = 0, A =a, \boldsymbol{C}= \boldsymbol{c}) \approx \beta _0 + \beta _1 a + \boldsymbol{\beta }'_2 \boldsymbol{c}$ and variance $\sigma ^2$ defines $\textrm{P}(\mathit{M} = \mathit{m}| \mathit{Y} = 0, \mathit{A} =\mathit{a}, \boldsymbol{C}= \boldsymbol{c})$.

This joint likelihood approach then expresses the mediator model among the cases as a function of the outcome odds and the mediator model among the controls:

17 $$\begin{aligned} \textrm{P}(\mathit{M}&= \mathit{m} | \mathit{Y} =1,\mathit{A} =\mathit{a}, \boldsymbol{C}= \boldsymbol{c}) \\&= \frac{\theta (a,m,\boldsymbol{c})\textrm{P}(\mathit{M}= \mathit{m} | \mathit{Y} =0,\mathit{a},\boldsymbol{c})}{\int \theta (a,m^*,\boldsymbol{c}) \textrm{P}(\mathit{M}= \mathit{m}^* | \mathit{Y} =0,\mathit{a},\boldsymbol{c}) \,\textrm{d} m^*}, \end{aligned}$$

where

$$\begin{aligned} \theta (a,m,c)= & {} \frac{\textrm{P}(\mathit{Y} =1|\mathit{A} =\mathit{a}, \mathit{M} = \mathit{m}, \boldsymbol{C}= \boldsymbol{c})}{\textrm{P}(\mathit{Y} =0|\mathit{A} =\mathit{a},\mathit{M} = \mathit{m}, \boldsymbol{C}= \boldsymbol{c})} \\= & {} \exp (\theta _0 + \theta _1 a + \theta _2 m + \theta _3 a m + \boldsymbol{\theta }_4' \boldsymbol{c}). \end{aligned}$$

The denominator in (17) is written as:

18 $$\begin{aligned} \int \theta (\mathit{a},m^*,\varvec{c}) \textrm{P}(\mathit{M}&= \mathit{m}^* | \mathit{Y} =0,\mathit{a},\varvec{c}) \,\textrm{d} \mathit{m}^* \\ &= \frac{\textrm{P}(\mathit{Y} =1|\mathit{a},\varvec{c})}{\textrm{P}(\mathit{Y} =0|\mathit{a},\varvec{c})} = \theta (\mathit{a},\varvec{c}), \end{aligned}$$

that is, it corresponds to the outcome odds given exposure and covariates. Hence

19 $$\begin{aligned} \textrm{P}(\mathit{Y} = \mathit{y} | \mathit{A} =\mathit{a}, \boldsymbol{C}= \boldsymbol{c}) = \frac{\theta (\mathit{a},\boldsymbol{c})^y}{1 + \theta (\mathit{a},\boldsymbol{c})}, \,\,y \in \{0,1\}, \end{aligned}$$

and the mediator model among the cases can be reexpressed as:

20 $$\begin{aligned} \textrm{P}(\mathit{M}= \mathit{m} | \mathit{Y} =1,\mathit{A} =\mathit{a}, \boldsymbol{C}= \boldsymbol{c}) = \frac{\theta (\mathit{a,m},\boldsymbol{c})\textrm{P}(\mathit{M}= \mathit{m} | \mathit{Y} =0,\mathit{a},\boldsymbol{c})}{\theta (\mathit{a},\boldsymbol{c})}. \end{aligned}$$

Combining these results, the following joint likelihood is obtained:

21 $$\begin{aligned} L_p = \prod _i \textrm{P}(\mathit{M} = \mathit{m}_{\mathit{i}}| \mathit{Y} = 0, \mathit{A =a}_\mathit{i}, \boldsymbol{C}= \boldsymbol{c}_{\mathit{i}}) \cdot \frac{\theta (\mathit{a}_{\mathit{i}},\mathit{m}_{\mathit{i}},\boldsymbol{c}_{\mathit{i}})^{\mathit{y}_{\mathit{i}}}}{1 + \theta (\mathit{a}_{\mathit{i}},\boldsymbol{c}_{\mathit{i}})}. \end{aligned}$$

Maximum likelihood estimators for the parameters of the mediator and outcome models are defined as arguments of the maxima of (21). Estimators for the natural effects ORs are then formed by substituting the parameters estimators in the approximate expressions (8-9) or (10-11).

This approach can be implemented using the R code provided by Satten et al. [17] (https://github.com/epstein-software/MediationCC).

### Simulation study

The objective of the simulation study was to assess the performance of the four approaches described previously in different case-control design scenarios, namely the : 1) approximate approach with the mediator model fitted among the controls only (*Approx_C*) ; 2) approximate approach with IPW (*Approx_IPW*) ; 3) exact approach with IPW (*Exact_IPW*) ; 4) unified likelihood approach (*Unified*). For reference, we also obtained results using the approximate and exact approaches which do not account for the case-control design (*Approx_Naive*, *Exact_Naive*). All six approaches were evaluated with respect to both continuous and binary mediators.

#### Data generation

We considered five scenarios in the continuous mediator case. In all scenarios, covariates $C_1$ and $C_2$ were generated independently as *Bernoulli*(0.5) and $\mathcal {N}(0,0.75^2)$ random variables, respectively. The binary exposure *A* was generated as a $Bernoulli(p_A)$, where $p_A =\text {expit}(-0.5 + 0.1 c_1 -0.15 c_2)$. The mediator *M* was generated as a $\mathcal {N}(\beta _0 + \beta _1 a + \beta _{21} c_1 + \beta _{22} c_2, 0.5^2)$, and the binary outcome *Y* was generated as a $Bernoulli(p_Y)$ with $p_Y =\text {expit}(\theta _0 + \theta _1 a + \theta _2 m \, + \theta _3 a m + \theta _{41} c_1 + \theta _{42} c_2)$. The mediator and outcome simulation parameters used for each scenario in the continuous mediator case are presented in Table A1 of Additional file 2.

As in the continuous mediator case, we considered five scenarios in the binary mediator case. In all scenarios, covariates $C_1$ and $C_2$ were generated independently as *Bernoulli*(0.5) and $\mathcal {N}(0,1)$, respectively. The binary exposure *A* was generated as a $Bernoulli(p_A)$, where $p_A =\text {expit}(-0.5 + 0.1 c_1 -0.15 c_2)$. The binary mediator *M* was generated as a $Bernoulli(p_M)$ with $p_M =\text {expit}(\beta _0 + \beta _1 a + \beta _{21} c_1 + \beta _{22} c_2)$, and the binary outcome as a $Bernoulli(p_Y)$ with $p_Y =\text {expit}(\theta _0 + \theta _1 a + \theta _2 m + \theta _3 a m + \theta _{41} c_1 + \theta _{42} c_2)$. The mediator and outcome simulation parameters used for each scenario in the binary mediator case are presented in Table A2 of Additional file 2.

We selected simulation scenarios to yield different marginal and conditional outcome prevalences (see Tables A3 and A4 of Additional file 2 for prevalences in the continuous and binary mediator cases, respectively). Values of the models’ parameters were also selected so to induce biased estimators of the regression parameters for the mediator model based on the selected samples. Specifically, in each scenario, a non-zero coefficient was specified for the mediator in the outcome model. Moreover, all variables (exposure and covariates) also had non-zero coefficients in the outcome model. It should be noted that the magnitude of selection bias induced is a function of the combined magnitude of these coefficients.

For both mediator types and each associated scenario, we constituted 1000 case-control samples from the corresponding population with an equal number of cases and controls, i.e., *n*/2 cases and *n*/2 controls for total sample sizes of $n=500$ and 1000.

#### Analysis

To provide an indication of the impact of the case-control design on the estimation of the mediator and outcome models’ parameters, we calculated the averages of the parameter values obtained across each set of 1000 case-control samples of size $n=1000$ according to different estimation strategies. First, by ignoring the design (*Naive*), second by using IPW in both the mediator and outcome models (*IPW*), third by estimating the mediator model using the controls only and the outcome model using all the sample (*Controls*), and fourth by using the joint likelihood (21) (*Unified*). For the first three strategies, a (possibly weighted) linear regression with the R function lm was used for the continuous mediator and a (possibly weighted) logistic regression using the R function glm was used for the binary variables. For each strategy and scenario, one-sample bilateral t-tests were performed to detect departures from the true values of the parameters.

We applied all six approaches to each case-control sample generated in order to estimate conditional natural direct and indirect effects on the OR scale. The implementation of the approximate approaches was done using the CMAverse package (available at https://bs1125.github.io/CMAverse/) with the R version 4.1.2. The implementation of the exact approaches was done using the ExactMed package version 0.3.0. All effects were computed by setting the covariates equal to the sample-specific means (that is, $\boldsymbol{C}=\boldsymbol{\bar{c}}$). For the approaches based on IPW, we used the true outcome prevalence (see Tables A3 and A4 in Additional file 2) as value for yprevalence. The bias, standard deviation (SD) and root mean squared error (RMSE) were calculated for each point estimator. Coverage probabilities of 95% CI estimators based on the delta method and percentile bootstrap were obtained for all approaches except for the unified likelihood approach where only the delta method is available from the R code provided by the authors. For each scenario, the true values of the NDE and NIE were computed using the true parameter values and population mean for the covariates.

For both mediator types we also investigated the impact of misspecifying the outcome prevalence $\pi$ on the natural effects estimates obtained from the approximate and exact approaches with IPW. Specifically, for each scenario, we considered a grid of values for $\pi$ which corresponded to a relative percentage of misspecification between -99% to 100%, by 5% from -95%. For each value of the prevalence’s grid, we computed the average natural effects based on the 1000 case-control samples of size $n=1000$ that were generated for each scenario. Results are presented visually next. Results for the approximate approach with the controls only and the unified approach, which both do not require the user to specify a value for $\pi$, are reported on corresponding figures for reference only.

## Results

The average estimated regression parameters for the continuous mediator case when $n=1000$ are presented in Table A1 of Additional file 3. From this table, we observe that for all scenarios, all coefficients estimated using IPW were globally in agreement with the true values. A few IPW averages showed small deviations from the true values, as highlighted by some smaller *p*-values. The parameters of the mediator model estimated from the controls only and using the joint likelihood of the unified approach were generally close. Important departures from the true values were observed for these two strategies, especially for Scenarios 2 and 5 where the ROA does not apply at least conditionally. For the outcome regression coefficients, only the intercept term was seen markedly affected by the design and only IPW correctly estimated it. In general, the unified approach slightly departed from the other approaches for the outcome model coefficients.

The natural effects results for the continuous mediator case when $n=1000$ are presented in Tables 1, 2, 3, 4, and 5. For Scenario 1, where the outcome is rare both marginally and conditionally, all approaches investigated, including the naive approaches that do not account for the case-control design, showed absolute relative biases less than 3% for all effects (NDE, NIE and TE). No significant undercoverage was observed throughout. For Scenario 2, where the outcome is rare marginally but is not conditionally rare in all quartiles of the mediator, both naive approaches yielded more important biases for the NDE and NIE. The approximate approach with IPW yielded relative biases below 10% for both the NDE and NIE but greater than 10% for the TE. The biases for the approximate approach with the mediator model fitted using the controls only were smaller, in absolute values, than for the approximate approach with IPW. The exact approach with IPW was the least biased for the NDE and NIE among all approaches compared. We observed that all approaches except the approximate naive and IPW approaches showed small biases for the TE. The results for Scenario 3, which features an outcome that is relatively common both marginally and conditionally, were similar to those obtained for Scenario 1. Although the outcome was relatively prevalent in Scenario 4 (marginal prevalence of 27.6%, conditional prevalences between 23% and 36%), the relative biases were small for all approaches and effects. In Scenario 5, the approximate approach with IPW yielded positive relative biases exceeding 10% for both NDE and NIE, largely impacting the TE (relative bias of 22.8%). The approximate approach with the mediator model fitted among the controls only yielded smaller biases in absolute value. For this approach, the positive bias seen for the NDE estimator coupled with the negative bias for the NIE estimator produced a TE estimator with only small bias. The exact approach with IPW yielded small relative biases for all effects in this scenario. Across all five scenarios, the unified approach often presented the smallest variability, however its performance in terms of RMSE depended on the amount of bias exhibited in a given scenario.

**Table 1:** Comparison of approaches for the estimation of natural effects on the odds ratio scale for Scenario 1 with a continuous mediator (based on 1000 data sets of size $n=1000$)

Effect	Approach	True value	Mean	Bias	Relative bias (%)	SD	RMSE	CP (%) delta	CP (%) boot
*NDE*	Approx_Naive	1.525	1.557	0.032	2.094	0.203	0.205	95.3	94.8
	Approx_C	1.525	1.558	0.032	2.129	0.204	0.207	95.2	95.0
	Approx_IPW	1.525	1.558	0.033	2.166	0.206	0.208	95.4	94.9
	Exact_Naive	1.525	1.541	0.016	1.031	0.206	0.206	95.1	95.3
	Exact_IPW	1.525	1.556	0.030	1.985	0.206	0.208	95.7	94.9
	Unified	1.525	1.558	0.033	2.135	0.204	0.207	95.2	-
*NIE*	Approx_Naive	1.064	1.073	0.009	0.838	0.046	0.047	95.9	94.2
	Approx_C	1.064	1.060	-0.004	-0.383	0.035	0.035	98.9	95.8
	Approx_IPW	1.064	1.062	-0.002	-0.158	0.037	0.037	98.6	95.7
	Exact_Naive	1.064	1.072	0.008	0.746	0.045	0.046	95.6	94.8
	Exact_IPW	1.064	1.062	-0.002	-0.181	0.037	0.037	98.7	95.7
	Unified	1.064	1.060	-0.004	-0.403	0.034	0.035	97.2	-
*TE*	Approx_Naive	1.623	1.669	0.046	2.830	0.214	0.219	95.5	95.2
	Approx_C	1.623	1.649	0.026	1.621	0.208	0.210	96.1	95.6
	Approx_IPW	1.623	1.653	0.031	1.883	0.211	0.213	96.1	95.4
	Exact_Naive	1.623	1.649	0.026	1.604	0.208	0.210	96.1	95.4
	Exact_IPW	1.623	1.650	0.027	1.674	0.210	0.212	96.2	95.4
	Unified	1.623	1.649	0.026	1.607	0.208	0.210	96.1	-

*boot* Bootstrap, *CP* Coverage probability, *NDE* Natural direct effect, *NIE* Natural indirect effect, *RMSE* Root mean squared error, *SD* Standard deviation, *TE* Total effect

**Table 2:** Comparison of approaches for the estimation of natural effects on the odds ratio scale for Scenario 2 with a continuous mediator (based on 1000 data sets of size $n=1000$)

Effect	Approach	True value	Mean	Bias	Relative bias (%)	SD	RMSE	CP (%) delta	CP (%) boot
*NDE*	Approx_Naive	1.622	1.840	0.218	13.445	0.285	0.358	88.1	86.6
	Approx_C	1.622	1.727	0.105	6.450	0.258	0.278	92.8	93.3
	Approx_IPW	1.622	1.752	0.130	8.028	0.266	0.296	92.4	91.6
	Exact_Naive	1.622	1.505	-0.117	-7.240	0.209	0.240	89.6	90.1
	Exact_IPW	1.622	1.652	0.030	1.835	0.238	0.239	94.7	94.7
	Unified	1.622	1.719	0.097	5.978	0.254	0.272	93.6	-
*NIE*	Approx_Naive	1.585	1.903	0.318	20.080	0.192	0.372	62.6	51.9
	Approx_C	1.585	1.547	-0.038	-2.385	0.108	0.114	98.7	92.0
	Approx_IPW	1.585	1.653	0.068	4.296	0.130	0.147	98.5	92.0
	Exact_Naive	1.585	1.737	0.152	9.611	0.124	0.197	79.6	76.5
	Exact_IPW	1.585	1.592	0.007	0.431	0.108	0.108	99.0	95.1
	Unified	1.585	1.525	-0.060	-3.797	0.095	0.113	88.1	-
*TE*	Approx_Naive	2.571	3.507	0.936	36.411	0.680	1.157	68.4	64.1
	Approx_C	2.571	2.659	0.089	3.460	0.361	0.372	98.1	94.8
	Approx_IPW	2.571	2.886	0.316	12.280	0.433	0.536	94.1	87.2
	Exact_Naive	2.571	2.605	0.034	1.332	0.346	0.348	94.9	94.3
	Exact_IPW	2.571	2.619	0.049	1.892	0.351	0.354	97.1	95.0
	Unified	2.571	2.610	0.040	1.540	0.347	0.349	95.2	-

*boot* Bootstrap, *CP* Coverage probability, *NDE* Natural direct effect, *NIE* Natural indirect effect, *RMSE* Root mean squared error, *SD* Standard deviation, *TE* Total effect

**Table 3:** Comparison of approaches for the estimation of natural effects on the odds ratio scale for Scenario 3 with a continuous mediator (based on 1000 data sets of size $n=1000$)

Effect	Approach	True value	Mean	Bias	Relative bias (%)	SD	RMSE	CP (%) delta	CP (%) boot
*NDE*	Approx_Naive	1.646	1.669	0.024	1.443	0.236	0.238	96.3	95.9
	Approx_C	1.646	1.675	0.029	1.772	0.241	0.243	96.6	95.7
	Approx_IPW	1.646	1.673	0.028	1.686	0.242	0.243	96.4	95.8
	Exact_Naive	1.646	1.662	0.017	1.018	0.245	0.245	95.6	95.3
	Exact_IPW	1.646	1.670	0.024	1.457	0.244	0.245	96.4	95.9
	Unified	1.646	1.676	0.030	1.844	0.241	0.243	96.3	-
*NIE*	Approx_Naive	1.150	1.157	0.007	0.606	0.094	0.095	94.4	94.0
	Approx_C	1.150	1.144	-0.005	-0.473	0.079	0.079	97.1	94.3
	Approx_IPW	1.150	1.151	0.001	0.100	0.087	0.087	96.8	93.9
	Exact_Naive	1.150	1.154	0.004	0.383	0.091	0.091	94.0	94.0
	Exact_IPW	1.150	1.149	0.000	-0.031	0.085	0.085	96.9	93.9
	Unified	1.150	1.143	-0.006	-0.548	0.078	0.078	95.0	-
*TE*	Approx_Naive	1.892	1.922	0.031	1.615	0.255	0.257	95.7	95.3
	Approx_C	1.892	1.908	0.016	0.850	0.245	0.245	95.8	95.0
	Approx_IPW	1.892	1.917	0.025	1.321	0.249	0.250	95.6	95.1
	Exact_Naive	1.892	1.908	0.016	0.843	0.244	0.245	95.3	95.5
	Exact_IPW	1.892	1.909	0.018	0.929	0.246	0.246	95.6	95.0
	Unified	1.892	1.908	0.016	0.851	0.244	0.245	95.3	-

*boot* Bootstrap, *CP* Coverage probability, *NDE* Natural direct effect, *NIE* Natural indirect effect, *RMSE* Root mean squared error, *SD* Standard deviation, *TE* Total effect

**Table 4:** Comparison of approaches for the estimation of natural effects on the odds ratio scale for Scenario 4 with a continuous mediator (based on 1000 data sets of size $n=1000$)

Effect	Approach	True value	Mean	Bias	Relative bias (%)	SD	RMSE	CP (%) delta	CP (%) boot
*NDE*	Approx_Naive	1.516	1.551	0.034	2.270	0.246	0.249	95.5	95.3
	Approx_C	1.516	1.554	0.037	2.468	0.250	0.252	95.6	94.8
	Approx_IPW	1.516	1.552	0.036	2.393	0.249	0.251	95.5	94.6
	Exact_Naive	1.516	1.543	0.027	1.761	0.253	0.254	95.4	95.0
	Exact_IPW	1.516	1.548	0.031	2.077	0.252	0.254	95.6	94.8
	Unified	1.516	1.554	0.038	2.497	0.249	0.252	95.4	-
*NIE*	Approx_Naive	1.105	1.111	0.006	0.564	0.112	0.113	94.6	94.4
	Approx_C	1.105	1.101	-0.004	-0.350	0.101	0.101	95.8	94.3
	Approx_IPW	1.105	1.107	0.002	0.204	0.108	0.108	95.2	94.2
	Exact_Naive	1.105	1.110	0.005	0.450	0.110	0.110	94.4	94.3
	Exact_IPW	1.105	1.106	0.001	0.111	0.106	0.106	95.2	93.9
	Unified	1.105	1.100	-0.005	-0.410	0.099	0.100	94.9	-
*TE*	Approx_Naive	1.675	1.707	0.032	1.914	0.222	0.224	95.1	94.3
	Approx_C	1.675	1.696	0.021	1.225	0.219	0.220	95.2	95.0
	Approx_IPW	1.675	1.703	0.028	1.688	0.222	0.223	95.0	94.4
	Exact_Naive	1.675	1.696	0.020	1.212	0.219	0.220	95.1	94.9
	Exact_IPW	1.675	1.696	0.021	1.242	0.220	0.221	95.2	95.2
	Unified	1.675	1.696	0.020	1.216	0.219	0.220	95.1	-

*boot* Bootstrap, *CP* Coverage probability, *NDE* Natural direct effect, *NIE* Natural indirect effect, *RMSE* Root mean squared error, *SD* Standard deviation, *TE* Total effect

**Table 5:** Comparison of approaches for the estimation of natural effects on the odds ratio scale for Scenario 5 with a continuous mediator (based on 1000 data sets of size $n=1000$)

Effect	Approach	True value	Mean	Bias	Relative bias (%)	SD	RMSE	CP (%) delta	CP (%) boot
*NDE*	Approx_Naive	1.415	1.609	0.194	13.708	0.274	0.336	91.3	90.2
	Approx_C	1.415	1.578	0.163	11.508	0.279	0.323	93.2	91.6
	Approx_IPW	1.415	1.599	0.184	12.967	0.276	0.332	91.7	90.8
	Exact_Naive	1.415	1.366	-0.049	-3.494	0.270	0.274	94.3	93.7
	Exact_IPW	1.415	1.445	0.030	2.127	0.271	0.273	95.7	95.1
	Unified	1.415	1.623	0.207	14.661	0.283	0.351	90.4	-
*NIE*	Approx_Naive	2.521	2.997	0.476	18.879	0.601	0.767	89.0	86.1
	Approx_C	2.521	2.363	-0.157	-6.237	0.294	0.334	98.2	92.7
	Approx_IPW	2.521	2.788	0.267	10.611	0.495	0.562	94.5	90.8
	Exact_Naive	2.521	2.707	0.186	7.379	0.409	0.450	92.3	92.4
	Exact_IPW	2.521	2.553	0.032	1.274	0.350	0.351	96.9	95.2
	Unified	2.521	2.259	-0.261	-10.369	0.246	0.359	85.3	-
*TE*	Approx_Naive	3.568	4.731	1.164	32.616	0.809	1.417	66.3	61.5
	Approx_C	3.568	3.678	0.111	3.100	0.496	0.509	97.3	94.5
	Approx_IPW	3.568	4.380	0.813	22.778	0.692	1.068	81.5	74.7
	Exact_Naive	3.568	3.618	0.050	1.405	0.479	0.482	95.8	95.1
	Exact_IPW	3.568	3.625	0.057	1.599	0.481	0.485	96.0	95.4
	Unified	3.568	3.622	0.055	1.529	0.480	0.483	95.7	-

*boot* Bootstrap, *CP* Coverage probability, *NDE* Natural direct effect, *NIE* Natural indirect effect, *RMSE* Root mean squared error, *SD* Standard deviation, *TE* Total effect

The results on the misspecification of the prevalence parameter $\pi$ when $n=1000$ are found in Figures A1-A5 in Additional file 3. The impact of the misspecification of $\pi$ on the natural direct and indirect effects results was generally minor for relative errors between -20% and 20% for the exact approach with IPW. When the parameter $\pi$ was importantly underestimated (corresponding to relative errors towards -99%), all the approaches accounting for the case-control design behaved similarly and could exhibit large departures from the true values of the effects. In this extreme case of prevalence misspecification, the $\theta _0$ parameter was largely underestimated by the exact approach with IPW in each scenario (results not shown), which yielded diminished differences between natural effects estimates obtained from the exact and approximate approaches. The exact approach was also seen affected when $\pi$ was importantly overestimated, most notably in Scenarios 4 and 5, but such relative errors implied that the posited outcome prevalences were larger than the exposure prevalences in these cases (corresponding to relative errors larger than 45%). For the TE, the approximate approach with IPW was noticeably more impacted than the exact approach with IPW. For this effect, the latter approach exhibited highly stable average estimates through the misspecification grid. This is not unexpected given that the exposure coefficient of an outcome logistic model in a non mediation analysis would not be impacted by the case-control design.

The average estimated regression coefficients for the binary mediator case when $n=1000$ are presented in Table A2 of Additional file 3. We observed greater differences between the regression coefficients of the mediator model estimated from the studied approaches in the binary mediator case than in the continuous one. Nonetheless, the qualitative conclusions regarding the impact of design on the estimated regression coefficients for the binary mediator case were similar to those for the continuous mediator case.

The natural effects results for the binary mediator case when $n=1000$ are presented in Tables 6, 7, 8, 9, and 10. For Scenario 1, where the outcome is rare both marginally and conditionally, all approaches except the approximate naive approach showed absolute relative biases less than 2% for all effects (NDE, NIE and TE). Some undercoverage was observed for the exact naive approach for the NIE. For Scenario 2, where the outcome is rare marginally but is not conditionally rare in strata defined by the levels of the binary exposure and binary mediator, all approaches except the exact approach with IPW yielded large relative bias for the NDE. The latter approach was also found with minimal relative bias for the NIE. For this effect, a large relative bias was observed for the approximate approach with the mediator model fitted using controls only while the relative bias for the approximate IPW approach was near but below 10%. The exact approach with IPW was also found particularly performant in terms of RMSE for the NDE and NIE in this scenario. The approximate approach with IPW yielded a relative bias exceeding 50% for the TE, while all other approaches accounting for the design produced a relative bias below 3% for this effect. The results for Scenario 3, which features an outcome that is relatively common both marginally and conditionally, were similar to those obtained for Scenario 1. However, while the relative biases were small throughout, some undercoverage was observed for the NIE in this scenario. Upon inspection of CIs for the NIE in this scenario (see Table 8), all approaches yielded larger average widths for the bootstrap CIs compared to the delta CIs, most notably the approaches accounting for the case-control design (results not shown). This would explain why the corresponding bootstrap CIs were found having better coverage than the delta CIs. In Scenarios 4 and 5, which both feature a common outcome, only the exact approach with IPW exhibited acceptable relative biases and coverage. The bias and undercoverage of other approaches were larger in Scenario 5 than in Scenario 4. Similar to what was observed in the continuous mediator case, the approximate approach with the mediator model fitted using the controls only and the unified approach did not exhibit relative bias issues for the TE in Scenario 5, unlike the approximate approach with IPW.

The impact of misspecification of $\pi$ on the natural effects estimates obtained from the IPW approaches was more visible in the binary mediator case with $n=1000$ (see Figures A6-A10 in Additional file 3). Nonetheless, the natural effects estimates from the exact approach with IPW were globally closer to the true effects over the middle of the misspecification grid for $\pi$ (relative errors between -20% and 20%) in all scenarios except in Scenario 1 where the approximate approach with IPW was uniformly closest.

**Table 6:** Comparison of approaches for the estimation of natural effects on the odds ratio scale for Scenario 1 with a binary mediator (based on 1000 data sets of size $n=1000$)

Effect	Approach	True value	Mean	Bias	Relative bias (%)	SD	RMSE	CP (%) delta	CP (%) boot
*NDE*	Approx_Naive	2.152	2.042	-0.110	-5.131	0.306	0.325	92.1	91.5
	Approx_C	2.152	2.179	0.027	1.233	0.306	0.307	94.5	93.3
	Approx_IPW	2.152	2.166	0.014	0.637	0.312	0.312	94.4	93.4
	Exact_Naive	2.152	2.193	0.041	1.923	0.304	0.307	93.9	93.4
	Exact_IPW	2.152	2.181	0.029	1.365	0.312	0.313	94.2	93.3
	Unified	2.152	2.177	0.024	1.137	0.305	0.306	94.0	-
*NIE*	Approx_Naive	1.047	1.044	-0.003	-0.274	0.032	0.032	89.6	93.7
	Approx_C	1.047	1.046	-0.001	-0.141	0.025	0.025	98.9	95.6
	Approx_IPW	1.047	1.047	0.000	0.008	0.027	0.027	98.8	96.2
	Exact_Naive	1.047	1.037	-0.010	-0.996	0.024	0.026	86.5	89.4
	Exact_IPW	1.047	1.046	-0.001	-0.136	0.025	0.025	98.9	95.4
	Unified	1.047	1.046	-0.001	-0.119	0.025	0.025	97.1	-
*TE*	Approx_Naive	2.254	2.133	-0.121	-5.378	0.328	0.349	92.0	91.5
	Approx_C	2.254	2.277	0.023	1.035	0.315	0.316	94.5	93.5
	Approx_IPW	2.254	2.267	0.013	0.594	0.323	0.323	94.6	93.4
	Exact_Naive	2.254	2.274	0.020	0.875	0.314	0.314	93.5	93.3
	Exact_IPW	2.254	2.280	0.026	1.174	0.322	0.323	94.5	93.4
	Unified	2.254	2.276	0.022	0.960	0.314	0.315	93.4	-

*boot* Bootstrap, *CP* Coverage probability, *NDE* Natural direct effect, *NIE* Natural indirect effect, *RMSE* Root mean squared error, *SD* Standard deviation, *TE* Total effect

**Table 7:** Comparison of approaches for the estimation of natural effects on the odds ratio scale for Scenario 2 with a binary mediator (based on 1000 data sets of size $n=1000$)

Effect	Approach	True value	Mean	Bias	Relative bias (%)	SD	RMSE	CP (%) delta	CP (%) boot
*NDE*	Approx_Naive	3.529	5.629	2.100	59.510	1.197	2.417	41.3	35.1
	Approx_C	3.529	4.890	1.361	38.566	1.011	1.696	68.7	64.0
	Approx_IPW	3.529	5.039	1.510	42.795	1.061	1.846	63.5	56.5
	Exact_Naive	3.529	2.733	-0.796	-22.544	0.399	0.890	55.0	57.4
	Exact_IPW	3.529	3.617	0.088	2.504	0.545	0.552	95.1	94.9
	Unified	3.529	4.897	1.368	38.767	1.011	1.701	71.5	-
*NIE*	Approx_Naive	1.460	2.486	1.026	70.293	0.254	1.057	0.0	0.0
	Approx_C	1.460	1.097	-0.362	-24.816	0.151	0.392	57.3	35.5
	Approx_IPW	1.460	1.581	0.121	8.281	0.151	0.194	97.2	87.6
	Exact_Naive	1.460	1.929	0.469	32.154	0.135	0.488	1.5	1.2
	Exact_IPW	1.460	1.472	0.013	0.869	0.116	0.117	99.0	95.6
	Unified	1.460	1.097	-0.362	-24.823	0.150	0.392	51.4	-
*TE*	Approx_Naive	5.151	14.039	8.888	172.557	3.546	9.570	0.6	0.5
	Approx_C	5.151	5.258	0.107	2.073	0.729	0.736	99.8	95.8
	Approx_IPW	5.151	7.899	2.748	53.351	1.496	3.129	61.6	32.7
	Exact_Naive	5.151	5.253	0.102	1.979	0.718	0.725	95.5	95.5
	Exact_IPW	5.151	5.301	0.150	2.911	0.740	0.755	97.8	95.3
	Unified	5.151	5.264	0.113	2.202	0.722	0.731	95.8	-

*boot* Bootstrap, *CP* Coverage probability, *NDE* Natural direct effect, *NIE* Natural indirect effect, *RMSE* Root mean squared error, *SD* Standard deviation, *TE* Total effect

**Table 8:** Comparison of approaches for the estimation of natural effects on the odds ratio scale for Scenario 3 with a binary mediator (based on 1000 data sets of size $n=1000$)

Effect	Approach	True value	Mean	Bias	Relative bias (%)	SD	RMSE	CP (%) delta	CP (%) boot
*NDE*	Approx_Naive	1.100	1.112	0.013	1.138	0.151	0.152	94.7	94.5
	Approx_C	1.100	1.120	0.020	1.844	0.152	0.153	94.8	94.2
	Approx_IPW	1.100	1.117	0.018	1.634	0.152	0.153	95.2	94.5
	Exact_Naive	1.100	1.092	-0.007	-0.638	0.144	0.144	94.8	94.3
	Exact_IPW	1.100	1.113	0.013	1.221	0.150	0.151	95.1	95.1
	Unified	1.100	1.119	0.019	1.736	0.152	0.153	94.9	-
*NIE*	Approx_Naive	0.939	0.964	0.025	2.704	0.016	0.030	61.1	64.6
	Approx_C	0.939	0.932	-0.007	-0.750	0.033	0.034	88.1	95.2
	Approx_IPW	0.939	0.942	0.003	0.303	0.028	0.028	87.1	94.4
	Exact_Naive	0.939	0.954	0.015	1.609	0.023	0.028	81.5	87.5
	Exact_IPW	0.939	0.937	-0.001	-0.156	0.031	0.031	88.9	95.3
	Unified	0.939	0.932	-0.007	-0.722	0.033	0.034	94.5	-
*TE*	Approx_Naive	1.032	1.072	0.040	3.830	0.143	0.149	93.9	94.4
	Approx_C	1.032	1.042	0.010	0.949	0.136	0.136	95.1	94.5
	Approx_IPW	1.032	1.051	0.019	1.842	0.139	0.140	95.1	94.4
	Exact_Naive	1.032	1.042	0.009	0.911	0.136	0.136	94.6	93.9
	Exact_IPW	1.032	1.042	0.010	0.956	0.136	0.137	95.4	94.1
	Unified	1.032	1.041	0.009	0.870	0.136	0.136	94.6	-

*boot* Bootstrap, *CP* Coverage probability, *NDE* Natural direct effect, *NIE* Natural indirect effect, *RMSE* Root mean squared error, *SD* Standard deviation, *TE* Total effect

**Table 9:** Comparison of approaches for the estimation of natural effects on the odds ratio scale for Scenario 4 with a binary mediator (based on 1000 data sets of size $n=1000$)

Effect	Approach	True value	Mean	Bias	Relative bias (%)	SD	RMSE	CP (%) delta	CP (%) boot
*NDE*	Approx_Naive	1.042	1.214	0.172	16.484	0.191	0.257	85.0	83.6
	Approx_C	1.042	1.151	0.109	10.480	0.196	0.224	93.0	91.9
	Approx_IPW	1.042	1.183	0.141	13.532	0.193	0.239	89.6	88.1
	Exact_Naive	1.042	0.965	-0.077	-7.398	0.213	0.227	90.0	91.0
	Exact_IPW	1.042	1.052	0.010	0.983	0.197	0.197	94.1	93.9
	Unified	1.042	1.153	0.111	10.699	0.196	0.226	92.9	-
*NIE*	Approx_Naive	1.927	1.701	-0.226	-11.736	0.154	0.274	72.1	66.3
	Approx_C	1.927	1.778	-0.149	-7.735	0.179	0.233	92.0	86.5
	Approx_IPW	1.927	1.745	-0.182	-9.452	0.166	0.246	86.2	79.0
	Exact_Naive	1.927	2.165	0.238	12.341	0.399	0.465	87.9	88.4
	Exact_IPW	1.927	1.958	0.031	1.592	0.265	0.266	94.3	93.0
	Unified	1.927	1.772	-0.155	-8.065	0.178	0.236	87.1	-
*TE*	Approx_Naive	2.008	2.052	0.044	2.174	0.292	0.295	94.7	94.2
	Approx_C	2.008	2.027	0.019	0.926	0.277	0.278	95.7	94.8
	Approx_IPW	2.008	2.048	0.040	1.999	0.287	0.290	95.0	94.3
	Exact_Naive	2.008	2.026	0.018	0.891	0.277	0.278	95.1	94.0
	Exact_IPW	2.008	2.026	0.018	0.892	0.277	0.278	95.6	95.0
	Unified	2.008	2.024	0.015	0.762	0.277	0.277	94.9	-

*boot* Bootstrap, *CP* Coverage probability, *NDE* Natural direct effect, *NIE* Natural indirect effect, *RMSE* Root mean squared error, *SD* Standard deviation, *TE* Total effect

**Table 10:** Comparison of approaches for the estimation of natural effects on the odds ratio scale for Scenario 5 with a binary mediator (based on 1000 data sets of size $n=1000$)

Effect	Approach	True value	Mean	Bias	Relative bias (%)	SD	RMSE	CP (%) delta	CP (%) boot
*NDE*	Approx_Naive	3.023	3.949	0.926	30.648	0.596	1.101	61.8	58.2
	Approx_C	3.023	3.808	0.785	25.979	0.596	0.986	71.7	69.1
	Approx_IPW	3.023	3.870	0.847	28.034	0.599	1.038	67.8	63.9
	Exact_Naive	3.023	2.529	-0.494	-16.346	0.560	0.747	83.3	85.6
	Exact_IPW	3.023	3.063	0.040	1.336	0.533	0.535	95.0	94.6
	Unified	3.023	3.811	0.788	26.058	0.598	0.989	72.8	-
*NIE*	Approx_Naive	2.213	1.838	-0.375	-16.948	0.134	0.398	23.5	21.5
	Approx_C	2.213	1.785	-0.428	-19.327	0.130	0.447	23.0	16.9
	Approx_IPW	2.213	1.842	-0.371	-16.752	0.135	0.395	29.7	25.6
	Exact_Naive	2.213	2.759	0.546	24.670	0.518	0.752	77.5	78.5
	Exact_IPW	2.213	2.238	0.025	1.148	0.266	0.268	96.6	94.9
	Unified	2.213	1.780	-0.433	-19.566	0.129	0.452	15.0	-
*TE*	Approx_Naive	6.690	7.233	0.543	8.124	1.053	1.185	93.8	92.3
	Approx_C	6.690	6.762	0.073	1.087	0.934	0.937	97.0	95.6
	Approx_IPW	6.690	7.099	0.409	6.119	1.022	1.100	95.2	94.1
	Exact_Naive	6.690	6.761	0.071	1.063	0.933	0.935	96.0	95.6
	Exact_IPW	6.690	6.773	0.083	1.239	0.942	0.945	96.6	95.5
	Unified	6.690	6.746	0.056	0.844	0.930	0.932	95.9	-

*boot* Bootstrap, *CP* Coverage probability, *NDE* Natural direct effect, *NIE* Natural indirect effect, *RMSE* Root mean squared error, *SD* Standard deviation, *TE* Total effect

The results when $n=500$ are presented in Additional file 4 (Tables A1-A5 for the continuous mediator case and Tables A6-A10 for the binary mediator case). The biases of the estimators were found generally larger when $n=500$ as when $n=1000$ and the bias patterns with respect to the studied estimators were roughly preserved. The coverage probabilities were found generally closer to the nominal value of 0.95 when $n=500$ as opposed to when $n=1000$. In the continuous mediator case, while the gain in using the exact approach with IPW was still visible from a bias perspective, it generally vanished when evaluated from a RMSE perspective. The exact approach with IPW still remained performant from a RMSE perspective in the binary mediator case.
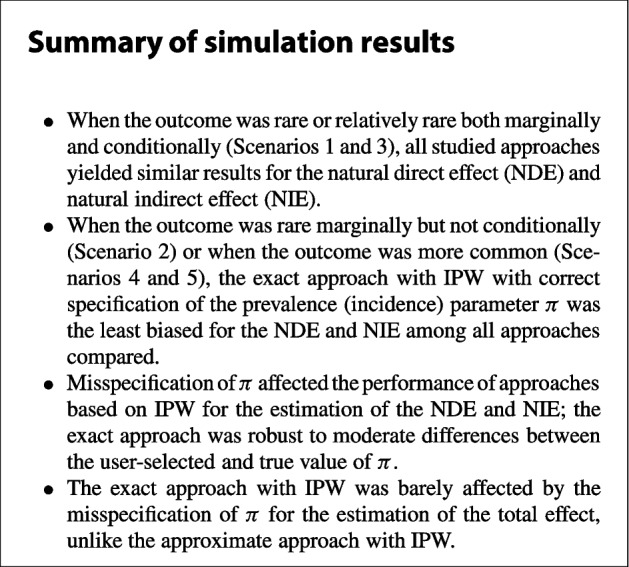


## Real data analysis

In this section, we apply the studied mediation analysis approaches to data from the PROVAQ study, a population-based case-control study on ovarian cancer [20]. It is well-established that oral contraceptive use lowers the risk of developing epithelial ovarian cancer [24]. However, the mechanisms of this protection are not clear. A long-standing model of ovarian carcinogenesis is the “incessant ovulation hypothesis”, which posits that ovulation entails repeated trauma and repair of the ovarian surface epithelium, and thus increases the possibility of DNA mutations leading to cancer initiation [25]. The contraceptive mechanism of most oral contraceptive types is ovulation suppression [26], thus the reduced risk of ovarian cancer with oral contraceptive use supports this hypothesis. However, it has been suggested that the magnitude of risk reduction with oral contraceptive use is stronger than that would be expected based on number of ovulations alone, and thus other mechanisms may be involved [27, 28]. In this application, the aim was to estimate the association between oral contraceptive use and ovarian cancer risk considering the natural mediation effects via the total number of ovulatory cycles over the lifetime.

Participants in the PROVAQ study were recruited from 2011 to 2016 and included Canadian citizens aged 18-79 years who resided in the greater Montreal area. Incident cases were identified in the major hospitals treating ovarian cancer in the study region while controls were selected from the Quebec electoral list and were frequency matched to cases by 5-year age group and Montreal region. Data were collected in an in-person interview. The final number of eligible participants was 498 cases of borderline ($n=134$) and invasive ($n=364$) ovarian cancers and 908 controls. A detailed description of the PROVAQ study was published previously [20]. The current analysis was restricted to cases of invasive ovarian cancer ($n=364$), which is the ovarian cancer type that has been consistently associated with oral contraceptive use.

The binary exposure variable was defined as the duration of oral contraceptive use, dichotomized as $\ge$ 10 years vs. < 10 years, the former level corresponding to the duration when a lower ovarian cancer risk is seen most consistently [24, 29]. The mediator, considered as a continuous variable, was defined as the lifetime number of ovulatory cycles, as calculated in the Cancer and Steroid Study (CASH) (equation 1) [30]. The binary outcome of case-control status represented incident invasive ovarian cancer cases and controls. Age and highest level of education attained were considered as potential confounding variables. Age was measured at diagnosis for cases and at interview for controls. Education was dichotomized as education level above high school or not. Lifetime number of ovulatory cycles could not be calculated for one control due to missing data, thus the final sample for the current analysis included 364 cases and 907 controls. Table 11 describes the cases and controls according to the variables used in our analysis.

All studied approaches were used to obtain conditional natural effects (NDE and NIE) assuming an interaction term between the mediator and exposure in the outcome regression model. As in the simulations, the conditioning values for the covariates were their average values in the sample (58.48 for age and 0.672 for education). In the exact and approximate approaches with IPW, we set $\pi =13.5/100\,000$, which corresponds to the annual incidence rate of ovarian cancer in Canada [31]. For the approximate approach with the controls only, we note that, since the controls were obtained through incidence density sampling, the equivalence (12) should hold exactly rather than approximately [32]. Moreover, in this context, conditions to interpret the ORs as instantaneous rate ratios would be the proportional-hazards assumption over the 5-year study period and the constant proportion of exposed (that is, long-term users of oral contraceptives) over that period [33].

Tables A1 and A2 of Additional file 5 show the estimated regression coefficients for the mediator and outcome models, respectively. The values shown in these tables correspond to those obtained using the exact approach with IPW (*Exact_IPW*). The point estimates are virtually the same as those obtained using the approximate approach with IPW (*Approx_IPW*), with very slight differences in the standard errors reported (results not shown). From Table A1 (Additional file 5), we see a strong association between the long-term use of oral contraceptives (exposure) and the lifetime number of ovulatory cycles (mediator), as expected. In the outcome model (see Table A2, Additional file 5), which included lifetime number of ovulatory cycles, long-term use of oral contraceptives was not found to be associated with ovarian cancer (outcome), either as a main effect term or as part of an interaction term with lifetime number of ovulatory cycles.

**Table 11:** Characteristics of the PROVAQ study sample

	Cases ($\boldsymbol{n = 364}$)	Controls ($\boldsymbol{n = 907}$)	Controls *Exposed* ($\boldsymbol{n = 242}$)	Controls *Unexposed* ($\boldsymbol{n = 665}$)
Duration of oral contraceptive use $\ge 10$ years, *n* (%)	61 (16.8 %)	242 (26.7 %)	—	—
Lifetime number of ovulatory cycles, mean (SD)	383.92 (111.07)	354.01 (122.20)	255.48 (98.93)	389.87 (109.53)
Age (years), mean (SD)	59.22 (11.35)	58.18 (12.62)	54.60 (11.20)	59.48 (12.86)
Highest education level > high school, *n* (%)	228 (62.64 %)	626 (69.02 %)	190 (78.51 %)	436 (65.56 %)

*SD* Standard deviation

**Table 12:** Estimated conditional total effect (*TE*) and natural direct effect (*NDE*) of long-term use of oral contraceptives on invasive ovarian cancer, with natural indirect effect (*NIE*) via lifetime number of ovulatory cycles

Effect	Approach	Estimate	SE	95% CI
*NDE*	Approx_Naive	0.655	0.174	0.389, 1.102
	Approx_C	0.657	0.170	0.396, 1.092
	Approx_IPW	0.650	0.152	0.411, 1.027
	Exact_Naive	0.659	0.171	0.396, 1.097
	Exact_IPW	0.650	0.152	0.411, 1.029
	Unified	0.654	0.158	0.407, 1.049
*NIE*	Approx_Naive	0.870	0.166	0.598, 1.264
	Approx_C	0.876	0.159	0.614, 1.249
	Approx_IPW	0.878	0.138	0.646, 1.194
	Exact_Naive	0.870	0.165	0.600, 1.262
	Exact_IPW	0.878	0.138	0.645, 1.196
	Unified	0.863	0.144	0.623, 1.196
*TE*	Approx_Naive	0.569	0.093	0.413, 0.785
	Approx_C	0.576	0.094	0.418, 0.794
	Exact_Naive	0.573	0.093	0.417, 0.788
	Approx_IPW	0.571	0.094	0.414, 0.787
	Exact_IPW	0.571	0.094	0.414, 0.787
	Unified	0.564	0.092	0.410, 0.776

*CI* Confidence interval, *SE* Standard error

The results of the mediation analysis are found in Table 12. The total effect and the natural direct and indirect effects were found similar across the approaches. We note that the results obtained with the exact and approximate approaches based on IPW (*Approx_IPW* and *Exact_IPW*) are practically identical. The TE ORs suggest that the risk of ovarian cancer at any time point is reduced with the long-term use of oral contraceptives (exact approach TE estimate: 0.571; 95% CI: 0.414 to 0.787). Natural effects estimates suggest a protective effect of long-term use of oral contraceptives that is both direct and indirect, but the results are not statistically significant. NDE ORs were found to be farther away from the null effect value ($OR=1$) than the NIE ORs, suggesting that the decrease in risk with long-term use of oral contraceptives is more important through pathways not involving the total number of ovulatory cycles over life.

Because the exposure-mediator interaction term included in the outcome model was not significant (*P*-value $=0.59$, see Table A2 of Additional file 5), a secondary mediation analysis which excluded that term from the outcome model was performed. The corresponding estimated regression coefficients and mediation effects are presented in Tables A3-A4 of Additional file 5, respectively. In this simpler outcome model, the exposure was found to be associated with the outcome (compare Tables A2 and A3 from Additional file 5). Some changes in the magnitude of the natural effects were observed : while the NDE ORs were again farther away from the null than the NIE ORs, the NDE and NIE ORs computed from this simpler model were respectively closer and farther to the null than when computed using the outcome model allowing for an exposure-mediator term. Moreover, significance was achieved for the NIE. Specifically, considering a long-term use of oral contraceptives in all the population, we would obtain near 20% risk reduction for ovarian cancer if lifetime number of ovulatory cycles were allowed to vary according to the long-term use of oral contraceptives or not (exact approach NIE estimate: 0.814 and 95% CI: 0.693 to 0.955).

## Discussion

In this article, we investigated the performance of different parametric regression-based approaches for the estimation of natural direct and indirect effects with a binary outcome and either a continuous or a binary mediator using case-control data. We have found that all approaches investigated yielded essentially similar results when the outcome was rare or relatively rare both marginally and conditionally. However, some differences between approaches were observed when the outcome was more common marginally and/or conditionally. In particular, only the exact approach with IPW was found to yield acceptable results in all of the simulation scenarios investigated. Regarding both approximate approaches by VanderWeele and collaborators, we have observed that the approximate approach that used the controls for the estimation of the mediator model yielded an estimator of the total effect that was less biased than when IPW was used. Indeed, while the estimated regression coefficients were appropriately corrected for the case-control design using IPW, the closed-form formulas used for the approximate NDE, NIE and TE estimands produced the biases observed for the natural effects estimators in some of the scenarios investigated. The unified approach proposed by Satten and collaborators was observed having similar issues with bias as the other approximate approaches for the estimation of the NDE and NIE. This unified approach was also found closest in behavior to the approximate approach with the controls only ; in particular, they agreed on the estimation of the regression parameters of the mediator model.

We have also investigated the impact of misspecifying the prevalence $\pi$ in the approximate and exact approaches that rely on a user-selected prevalence value (IPW). In our simulations, in which the relative misspecification of $\pi$ was allowed to range between $-99\%$ and $100\%$, we observed that the misspecification of $\pi$ was less of a concern than the approximate or exact nature of the natural effects estimands when the misspecification was moderate. When the prevalence was importantly underestimated, all studied approaches were found to behave similarly. This is an interesting observation since one can thus view the “controls only” strategy as making *implicitly* an extreme choice for the user provided prevalence parameter $\pi$. Indeed fitting the mediator model with controls only is equivalent to setting the yprevalence parameter to zero, in which situation the cases receive null weights when fitting this model.

In this work, we considered the exact estimators with IPW to allow for direct comparisons with the approximate approach with IPW and provide an evaluation of the ExactMed R package for the estimation of natural effects with case-control data. However, other strategies for the estimation of the regression parameters to be used in exact estimators could be envisaged in this context. In the case of a binary mediator, Doretti et al. [14] proposed M-estimation or maximum likelihood estimation for the simultaneous estimation of the regression coefficients of the mediator and outcome models, but nevertheless assume the population prevalence $\pi$ known for implementing the correction related to the intercept coefficient of the outcome model. These authors found that such approaches yield estimators that both properly adjust for the case-control design and exhibit increased efficiency as compared to IPW.

Lastly, we believe worth raising the fact that, in the context of case-control study designs, the choice of the conditioning values of the covariates used for computing the conditional natural effects may produce interpretation issues. As pointed out in VanderWeele and Tchetgen Tchetgen [32], using the empirical means of the covariates $\bar{\boldsymbol{C}}$ found in a selected sample may not well approximate the population averages *E*[***C***] (even with a large sample). Currently, and to the best of our understanding, this is the default procedure in packages CMAverse and ExactMed. Therefore, to the extent that $\bar{\boldsymbol{C}}$ is not a convergent estimator for *E*[***C***], this has for consequence that the studied conditional natural effects estimators do not exactly target the correct estimands, which are conceptualized to be defined based on the population means. While this was found practically inconsequential in the simulations, it could be otherwise in other sets-up. Automatically computing the conditional natural effects with the covariates means corrected using IPW could provide a sensible upgrade when a case-control option is used.

## Conclusion

We have brought additional insights on existing regression-based approaches for estimating natural direct and indirect effects for a binary outcome and a continuous or binary mediator using data from case-control study designs. Studied estimators rely either on the ROA or knowledge of the outcome prevalence (incidence) in the population, or both. Given that the former can be difficult to assess with respect to all relevant strata formed by the conditioning variables (exposure and mediator) of the outcome model and the latter difficult to specify exactly, we recommend evaluating the robustness of natural effects estimates by use of different estimators. However, approximate mediation approaches should be avoided or regarded with caution in situations where a violation of the ROA applies or is expected. As was found in the context of cohort study designs, the exact estimators investigated herein circumvented the difficulties associated with this assumption, and are thus to be favored in the previous situations. Nonetheless, the performance of these estimators, as the approximate estimators based on IPW, depends on the correct specification of the outcome prevalence (incidence) parameter $\pi$ and we cannot eliminate the possibility that the exact estimators yield worse results than the approximate ones, even for moderate misspecification of $\pi$. Sensitivity analyses with respect to the specification of $\pi$ should be performed whenever there is significant uncertainty regarding the outcome prevalence (incidence) in the population.

As a final remark, the exact approaches studied herein have not been yet evaluated for mediation analysis with multiple mediators based on cohort data. Considering extant knowledge for multiple mediation analysis (e.g., [34]), it is reasonable to believe that these approaches for a single mediator could be used separately on each mediator when they are conditionally independent given the covariates in the population, and that IPW could be used to account for the design if implemented on case-control data. This interesting line of inquiry should be evaluated in future research.

### Supplementary Information

**Additional file 1.** Variance of natural effects estimators. We explain how the variance of natural effects estimators and the confidence intervals for these effects were obtained via the exact approaches (naive and IPW) for both the continuous and the binary mediator cases.

**Additional file 2.** Details on the simulation scenarios. We present the true values of the parameters used in the five simulation scenarios for each type of mediator (continuous and binary). We also present the corresponding prevalences for the outcome and exposure.

**Additional file 3.** Additional simulation results when *n *= 1000. We present the average regression parameters estimated using the studied strategies in our main simulations with *n *= 1000. We also present figures showing the impact of the misspecification of the prevalence parameter $\pi$ on the performance of the exact and approximate approaches with IPW in the simulations with *n *= 1000.

**Additional file 4.** Simulation results with *n *= 500. We present the natural effects estimates obtained with the studied mediation approaches with generated samples of size *n *= 500.

**Additional file 5.** Additional results for the real-data analysis. We present mediation analysis results based on the data from the PROVAQ study, including the estimated mediator and outcome models for the main analysis as well as results corresponding to the simpler outcome model which omits the interaction term between the exposure and mediator.

## Data Availability

The data from PROVAQ that support these findings are available from the PROVAQ PI (AK) upon reasonable request and institutional approval.

## Abbreviations

boot: Bootstrap
CI: Confidence interval
CP: Coverage probability
IPW: Inverse probability weighting
NDE: Natural direct effect
NIE: Natural indirect effect
PROVAQ: PRevention of OVArian Cancer in Quebec
RMSE: Root mean squared error
ROA: Rare outcome assumption
SD: Standard deviation
SE: Standard error
TE: Total effect
