# Supplementary material for: An empirical evaluation of approximate and exact regression-based causal mediation approaches for a binary outcome and a continuous or a binary mediator for case-control study designs
Source: BMC Med Res Methodol. 2024 Mar 20;24:72. doi: 10.1186/s12874-024-02156-y (PMC10953265; doi:10.1186/s12874-024-02156-y)
Supplement: Supplementary file 1 — Additional file 1. Variance of natural effects estimators. We explain how the variance of natural effects estimators and the confidence intervals for these effects were obtained via the exact approaches (naive and IPW) for both the continuous and the binary mediator cases. [file 12874_2024_2156_MOESM1_ESM.pdf]

We present information on interval estimation of natural effects (log) odds ratios (ORs) based on the exact approaches (naive and IPW) in the binary and continuous mediator cases.

For both types of mediators, we use the first-order multivariate delta method [1] to construct the 95% confidence intervals (CIs) for the natural effects ORs (Equations (1-2) in the main text). The standard errors for $\ln(\widehat{OR}^{NDE}_{a,a^*}(\boldsymbol{c}))$, $\ln(\widehat{OR}^{NIE}_{a,a^*}(\boldsymbol{c}))$ and $\ln(\widehat{OR}^{TE}_{a,a^*}(\boldsymbol{c}))$ are expressed according to the following approximate formulas (also used in [2]):

$$\begin{aligned}
\mathrm{se}(\ln(\widehat{OR}^{NDE}_{a,a^*}(\boldsymbol{c}))) &\approx \sqrt{\nabla(\ln(\widehat{OR}^{NDE}_{a,a^*}(\boldsymbol{c})))' \cdot \boldsymbol{\Sigma} \cdot \nabla(\ln(\widehat{OR}^{NDE}_{a,a^*}(\boldsymbol{c})))},\\
\mathrm{se}(\ln(\widehat{OR}^{NIE}_{a,a^*}(\boldsymbol{c}))) &\approx \sqrt{\nabla(\ln(\widehat{OR}^{NIE}_{a,a^*}(\boldsymbol{c})))' \cdot \boldsymbol{\Sigma} \cdot \nabla(\ln(\widehat{OR}^{NIE}_{a,a^*}(\boldsymbol{c})))},\\
\mathrm{se}(\ln(\widehat{OR}^{TE}_{a,a^*}(\boldsymbol{c}))) &\approx \sqrt{\nabla(\ln(\widehat{OR}^{TE}_{a,a^*}(\boldsymbol{c})))' \cdot \boldsymbol{\Sigma} \cdot \nabla(\ln(\widehat{OR}^{TE}_{a,a^*}(\boldsymbol{c})))}.
\end{aligned}$$

In these formulas, $\boldsymbol{\Sigma} = diag\{\boldsymbol{\Sigma}_{\hat{\boldsymbol{\beta}}}, \boldsymbol{\Sigma}_{\hat{\boldsymbol{\theta}}}\}$ is a block matrix, $\boldsymbol{\Sigma}_{\hat{\boldsymbol{\beta}}}$ and $\boldsymbol{\Sigma}_{\hat{\boldsymbol{\theta}}}$ are the covariance matrices for the vectors $\hat{\boldsymbol{\beta}}$ and $\hat{\boldsymbol{\theta}}$ respectively. We distinguish between estimators $\hat{\boldsymbol{\beta}}$ and $\hat{\boldsymbol{\theta}}$ defined under the exact approach without weighting (Exact_Naive) and those defined under the exact approach with weighting (Exact_IPW). The naive exact approach considers the standard estimators for the matrices $\boldsymbol{\Sigma}_{\hat{\boldsymbol{\beta}}}$ and $\boldsymbol{\Sigma}_{\hat{\boldsymbol{\theta}}}$ (e.g., inverse of the Fisher information matrix evaluated at the fitted coefficients for a logistic model). The exact approach with IPW instead uses robust estimators for the variance-covariance matrices since observations are being weighted (HC3 type covariance matrix estimator, which is the default type in the function vcovHC of R package `sandwich`). The expressions, $\nabla(\ln(\widehat{OR}^{NDE}_{a,a^*}(\boldsymbol{c})))$, $\nabla(\ln(\widehat{OR}^{NIE}_{a,a^*}(\boldsymbol{c})))$ and $\nabla(\ln(\widehat{OR}^{TE}_{a,a^*}(\boldsymbol{c})))$ correspond to the gradients of $\ln(OR^{NDE}_{a,a^*}(\boldsymbol{c}))$, $\ln(OR^{NIE}_{a,a^*}(\boldsymbol{c}))$ and $\ln(OR^{TE}_{a,a^*}(\boldsymbol{c}))$, where the last one is obtained by the relation:

$$\nabla(\ln(\widehat{OR}^{TE}_{a,a^*}(\boldsymbol{c}))) = \nabla(\ln(\widehat{OR}^{NDE}_{a,a^*}(\boldsymbol{c}))) + \nabla(\ln(\widehat{OR}^{NIE}_{a,a^*}(\boldsymbol{c}))).$$

These gradients are evaluated at the estimated values of the regression parameters, either obtained from a naive or a IPW strategy.

It must be noted that in the continuous mediator case, obtaining the counterfactual probabilities involved in the definitions of effects, as well as the corresponding gradients, requires numerically evaluating the integral involved in the different expressions. We refer readers to Samoilenko and Lefebvre [3] and Samoilenko and Lefebvre [4] for the detailed gradient expressions in the binary and continuous mediator cases, respectively.

Finally, since $\ln(\widehat{OR}^{NDE}_{a,a^*}(\boldsymbol{c}))$, $\ln(\widehat{OR}^{NIE}_{a,a^*}(\boldsymbol{c}))$ and $\ln(\widehat{OR}^{TE}_{a,a^*}(\boldsymbol{c}))$ are approximately normally distributed, 95% CIs for the natural effects ORs are given by:

$$\widehat{OR}^{NDE}_{a,a^*}(\boldsymbol{c}) \cdot \exp(\pm\Phi^{-1}(0.975) \cdot \mathrm{se}(\ln(\widehat{OR}^{NDE}_{a,a^*}(\boldsymbol{c})))),$$
$$\widehat{OR}^{NIE}_{a,a^*}(\boldsymbol{c}) \cdot \exp(\pm\Phi^{-1}(0.975) \cdot \mathrm{se}(\ln(\widehat{OR}^{NIE}_{a,a^*}(\boldsymbol{c})))),$$
$$\widehat{OR}^{TE}_{a,a^*}(\boldsymbol{c}) \cdot \exp(\pm\Phi^{-1}(0.975) \cdot \mathrm{se}(\ln(\widehat{OR}^{TE}_{a,a^*}(\boldsymbol{c})))).$$

References

1. Casella, G., Berger, R.L.: Statistical Inference vol. 2. Duxbury Pacific Grove, CA, (2002).
2. VanderWeele, T.J.: Explanation in Causal Inference: Methods for Mediation and Interaction. Oxford University Press, New York, NY (2015).
3. Samoilenko, M., Lefebvre, G.: Parametric-regression-based causal mediation analysis of binary outcomes and binary mediators: Moving beyond the rareness or commonness of the outcome. American Journal of Epidemiology 190(9), 1846–1858 (2021).
4. Samoilenko, M., Lefebvre, G.: An exact regression-based approach for the estimation of natural direct and indirect effects with a binary outcome and a continuous mediator. Statistics in Medicine 42(3), 353–387 (2023).
