# Supplementary material for: An empirical evaluation of approximate and exact regression-based causal mediation approaches for a binary outcome and a continuous or a binary mediator for case-control study designs
Source: BMC Med Res Methodol. 2024 Mar 20;24:72. doi: 10.1186/s12874-024-02156-y (PMC10953265; doi:10.1186/s12874-024-02156-y)
Supplement: Supplementary file 2 — Additional file 2. Details on the simulation scenarios. We present the true values of the parameters used in the five simulation scenarios for each type of mediator (continuous and binary). We also present the corresponding prevalences for the outcome and exposure. [file 12874_2024_2156_MOESM2_ESM.pdf]

Table A1 Simulation study for the continuous mediator case: regression coefficients of the mediator (β) and outcome (θ) models for each scenario.

	β_0	β_1	β_{21}	β_{22}	θ_0	θ_1	θ_2	θ_3	θ_{41}	θ_{42}
Scenario 1	0.1	0.2	0.1	0.2	-3.0	0.4	0.2	0.11	0.1	-0.3
Scenario 2	-0.1	0.3	0.1	0.2	-2.75	0.4	1.2	0.45	-0.2	-0.3
Scenario 3	0.1	0.4	0.1	0.2	-2.22	0.5	0.35	0	0.2	-0.1
Scenario 4	0.1	0.5	0.1	0.2	-1.3	0.4	0.1	0.1	0.2	-0.1
Scenario 5	0.1	0.8	0.1	0.2	-1.6	0.1	0.2	1.05	0.2	-0.1

Table A2 Simulation study for the binary mediator case: regression coefficients of the mediator (β) and outcome (θ) models for each scenario.

	β_0	β_1	β_{21}	β_{22}	θ_0	θ_1	θ_2	θ_3	θ_{41}	θ_{42}
Scenario 1	-2.197	0.811	0.2	0.25	-3.476	0.889	1.034	-0.644	0.25	0.2
Scenario 2	-2.197	0.811	0.2	0.25	-3.476	0.889	1.034	1.553	0.25	0.2
Scenario 3	-2.197	0.811	0.2	0.25	-1.901	0.166	0.086	-0.793	0.25	0.2
Scenario 4	0.201	-2.643	0.2	0.25	-1.153	0.489	-0.582	-1.068	0.25	0.2
Scenario 5	0.201	-2.643	0.2	0.25	-1.992	1.792	-0.450	-1.547	0.25	0.2

Table A3 Prevalences of exposure (A) and outcome (Y)†. Conditional prevalences for the outcome are obtained for each quartile of the continuous mediator (by row, Q_1 to Q_4, resp.) and each level of exposure ($A = 0, 1$).

Scenario	A	Y	Conditional Y
1	0.39	0.064	(0.05, 0.05, 0.05, 0.05;<br>0.07, 0.08, 0.08, 0.09)
2	0.39	0.091	(0.03, 0.05, 0.07, 0.11;<br>0.04, 0.07, 0.12, 0.24)
3	0.39	0.146	(0.10, 0.11, 0.12, 0.14;<br>0.15, 0.17, 0.19, 0.22)
4	0.39	0.276	(0.23, 0.23, 0.24, 0.25;<br>0.30, 0.32, 0.34, 0.36)
5	0.39	0.290	(0.17, 0.19, 0.20, 0.22;<br>0.17, 0.26, 0.37, 0.56)

† : approximated using 10^7 observations generated for each scenario

Table A4 Prevalences of exposure (A), mediator (M) and outcome (Y)†. Conditional prevalences for the outcome are obtained for each strata formed by the levels of binary exposure and binary mediator††.

Scenario	A	M	Y	Conditional Y
1	0.39	0.154	0.059	(0.035, 0.097, 0.079, 0.117)
2	0.39	0.154	0.095	(0.035, 0.097, 0.079, 0.539)
3	0.39	0.154	0.150	(0.147, 0.166, 0.166, 0.094)
4	0.39	0.386	0.262	(0.261, 0.174, 0.366, 0.107)
5	0.39	0.386	0.241	(0.133, 0.094, 0.477, 0.118)

† : approximated using 10^7 observations for each scenario. †† : $(A = 0, M = 0;\ A = 0, M = 1;\ A = 1, M = 0;\ A = 1, M = 1)$
