# Supplementary material for: An empirical evaluation of approximate and exact regression-based causal mediation approaches for a binary outcome and a continuous or a binary mediator for case-control study designs
Source: BMC Med Res Methodol. 2024 Mar 20;24:72. doi: 10.1186/s12874-024-02156-y (PMC10953265; doi:10.1186/s12874-024-02156-y)
Supplement: Supplementary file 3 — Additional file 3. Additional simulation results when n = 1000. We present the average regression parameters estimated using the studied strategies in our main simulations with n = 1000. We also present figures showing the impact of the misspecification of the prevalence parameter $\pi$π on the performance of the exact and approximate approaches with IPW in the simulations with n = 1000. [file 12874_2024_2156_MOESM3_ESM.pdf]

1.1 Average estimated regression parameters

Tables A1 and A2 present the average estimated regression parameters for the continuous and binary mediator cases, respectively. In these tables, the strategy refers to the way the regression parameters were estimated. Note that the results for the Naive and IPW strategies coincide exactly with results obtained for the parameters using the CMAverse and ExactMed packages. Moreover, regression parameters obtained from the Controls strategy coincide exactly with the parameters returned from the CMAverse package when this option is used. P-values from one-sample bilateral t-tests are also provided, where, for each test, the true value of the parameter is used for the hypothesized value of the mean.

1.2 Impact of prevalence misspecification

The impact of prevalence misspecification π on the natural effects estimates are presented in Figures A1 to A10.

Table A1 Average estimated regression coefficients (p-value from t-test) in the continuous mediator case. Results based on 1000 data sets of size $n = 1000$.

	Strategy	β_0	β_1	β_{21}	β_{22}	σ	θ_0	θ_1	θ_2	θ_3	θ_{41}	θ_{42}
Sc. 1	True	0.100	0.200	0.100	0.200	0.500	-3.000	0.400	0.200	0.110	0.100	-0.300
	Naive	0.119 ($2 \cdot 10^{-16}$)	0.218 ($2 \cdot 10^{-16}$)	0.101 (0.373)	0.195 ($1 \cdot 10^{-14}$)	0.500 (0.336)	-0.322 ($2 \cdot 10^{-16}$)	0.408 (0.105)	0.205 (0.378)	0.115 (0.603)	0.100 (0.952)	-0.303 (0.385)
	Controls†	0.097 (0.013)	0.196 (0.009)	0.100 (0.968)	0.198 (0.018)	0.499 (0.111)	-0.322 ($2 \cdot 10^{-16}$)	0.408 (0.105)	0.205 (0.378)	0.115 (0.603)	0.100 (0.952)	-0.303 (0.385)
	IPW	0.100 (0.710)	0.200 (0.945)	0.100 (0.824)	0.197 ($5 \cdot 10^{-4}$)	0.499 (0.006)	-3.004 (0.191)	0.407 (0.115)	0.205 (0.365)	0.116 (0.479)	0.100 (0.958)	-0.303 (0.266)
	Unified	0.098 (0.026)	0.196 (0.003)	0.099 (0.290)	0.198 (0.022)	0.498 ($5 \cdot 10^{-9}$)	-0.322 ($2 \cdot 10^{-16}$)	0.408 (0.098)	0.205 (0.366)	0.114 (0.640)	0.100 (0.985)	-0.302 (0.417)
Sc. 2	True	-0.100	0.300	0.100	0.200	0.500	-2.750	0.400	1.200	0.450	-0.200	-0.300
	Naive	-0.003 ($2 \cdot 10^{-16}$)	0.385 ($2 \cdot 10^{-16}$)	0.099 (0.212)	0.199 (0.045)	0.514 ($2 \cdot 10^{-16}$)	-0.457 ($2 \cdot 10^{-16}$)	0.409 (0.077)	1.214 (0.025)	0.444 (0.495)	-0.199 (0.888)	-0.308 (0.011)
	Controls†	-0.118 ($2 \cdot 10^{-16}$)	0.265 ($2 \cdot 10^{-16}$)	0.100 (0.907)	0.202 (0.098)	0.491 ($2 \cdot 10^{-16}$)	-0.457 ($2 \cdot 10^{-16}$)	0.409 (0.077)	1.214 (0.025)	0.444 (0.495)	-0.199 (0.888)	-0.308 (0.011)
	IPW	-0.100 (0.966)	0.301 (0.632)	0.099 (0.632)	0.201 (0.247)	0.499 (0.051)	-2.759 (0.004)	0.404 (0.479)	1.217 (0.010)	0.459 (0.373)	-0.196 (0.365)	-0.311 (0.001)
	Unified	-0.119 ($2 \cdot 10^{-16}$)	0.264 ($2 \cdot 10^{-16}$)	0.103 (0.005)	0.200 (0.987)	0.486 ($2 \cdot 10^{-16}$)	-0.460 ($2 \cdot 10^{-16}$)	0.431 ($4 \cdot 10^{-10}$)	1.245 ($1 \cdot 10^{-12}$)	0.364 ($2 \cdot 10^{-16}$)	-0.198 (0.674)	-0.307 (0.019)
Sc. 3	True	0.100	0.400	0.100	0.200	0.500	-2.220	0.500	0.350	0.000	0.200	-0.100
	Naive	0.128 ($2 \cdot 10^{-16}$)	0.405 ($8 \cdot 10^{-8}$)	0.101 (0.327)	0.199 (0.071)	0.500 (0.819)	-0.454 ($2 \cdot 10^{-16}$)	0.502 (0.668)	0.354 (0.488)	-0.003 (0.695)	0.198 (0.606)	-0.098 (0.487)
	Controls†	0.093 ($1 \cdot 10^{-9}$)	0.392 ($5 \cdot 10^{-8}$)	0.097 (0.025)	0.198 (0.076)	0.498 ($8 \cdot 10^{-5}$)	-0.454 ($2 \cdot 10^{-16}$)	0.502 (0.668)	0.354 (0.488)	-0.003 (0.695)	0.198 (0.606)	-0.098 (0.487)
	IPW	0.102 (0.091)	0.399 (0.429)	0.099 (0.457)	0.198 (0.041)	0.498 ($5 \cdot 10^{-4}$)	-2.221 (0.691)	0.501 (0.782)	0.355 (0.407)	-0.001 (0.901)	0.198 (0.645)	-0.098 (0.591)
	Unified	0.093 ($5 \cdot 10^{-11}$)	0.392 ($2 \cdot 10^{-7}$)	0.096 ($9 \cdot 10^{-5}$)	0.199 (0.230)	0.497 ($2 \cdot 10^{-16}$)	-0.454 ($2 \cdot 10^{-16}$)	0.504 (0.483)	0.355 (0.396)	-0.006 (0.430)	0.198 (0.608)	-0.098 (0.447)
Sc. 4	True	0.100	0.500	0.100	0.200	0.500	-1.300	0.400	0.100	0.100	0.200	-0.100
	Naive	0.105 ($1 \cdot 10^{-10}$)	0.506 ($6 \cdot 10^{-9}$)	0.101 (0.301)	0.199 (0.099)	0.499 (0.102)	-0.338 ($2 \cdot 10^{-16}$)	0.407 (0.237)	0.106 (0.249)	0.093 (0.347)	0.202 (0.579)	-0.107 (0.021)
	Controls†	0.094 ($6 \cdot 10^{-8}$)	0.490 ($2 \cdot 10^{-11}$)	0.100 (0.892)	0.200 (0.736)	0.500 (0.329)	-0.338 ($2 \cdot 10^{-16}$)	0.407 (0.237)	0.106 (0.249)	0.093 (0.347)	0.202 (0.579)	-0.107 (0.021)
	IPW	0.100 (0.821)	0.500 (0.832)	0.101 (0.371)	0.199 (0.220)	0.499 (0.117)	-1.304 (0.129)	0.407 (0.219)	0.106 (0.247)	0.092 (0.340)	0.203 (0.526)	-0.107 (0.020)
	Unified	0.095 ($4 \cdot 10^{-8}$)	0.490 ($4 \cdot 10^{-12}$)	0.099 (0.330)	0.199 (0.342)	0.498 ($4 \cdot 10^{-11}$)	-0.338 ($2 \cdot 10^{-16}$)	0.408 (0.189)	0.106 (0.230)	0.091 (0.268)	0.202 (0.619)	-0.107 (0.023)
Sc. 5	True	0.100	0.800	0.100	0.200	0.500	-1.600	0.100	0.200	1.050	0.200	-0.100
	Naive	0.109 ($2 \cdot 10^{-16}$)	0.853 ($2 \cdot 10^{-16}$)	0.100 (0.631)	0.200 (0.577)	0.499 ($3 \cdot 10^{-4}$)	-0.711 ($2 \cdot 10^{-16}$)	0.094 (0.471)	0.196 (0.428)	1.069 (0.034)	0.205 (0.209)	-0.101 (0.679)
	Controls†	0.094 ($4 \cdot 10^{-8}$)	0.680 ($2 \cdot 10^{-16}$)	0.094 ($8 \cdot 10^{-6}$)	0.198 (0.073)	0.493 ($2 \cdot 10^{-16}$)	-0.711 ($2 \cdot 10^{-16}$)	0.094 (0.471)	0.196 (0.428)	1.069 (0.034)	0.205 (0.209)	-0.101 (0.679)
	IPW	0.100 (0.803)	0.800 (0.944)	0.101 (0.558)	0.201 (0.173)	0.499 (0.154)	-1.604 (0.219)	0.093 (0.408)	0.196 (0.443)	1.071 (0.229)	0.204 (0.370)	-0.102 (0.618)
	Unified	0.096 ($1 \cdot 10^{-4}$)	0.680 ($2 \cdot 10^{-16}$)	0.089 ($2 \cdot 10^{-16}$)	0.197 ($2 \cdot 10^{-7}$)	0.489 ($2 \cdot 10^{-16}$)	-0.711 ($2 \cdot 10^{-16}$)	0.157 ($8 \cdot 10^{-15}$)	0.204 (0.544)	0.996 ($4 \cdot 10^{-10}$)	0.203 (0.556)	-0.099 (0.702)

† : β coefficients estimated based on the controls only and θ coefficients estimated based on all the sample (cases and controls). **Note**: p-values smaller than $2 \cdot 10^{-16}$ are shown as $2 \cdot 10^{-16}$.

Table A2 Average estimated regression coefficients (p-value from t-test) in the binary mediator case. Results based on 1000 data sets of size $n = 1000$.

	Strategy	β_0	β_1	β_{21}	β_{22}	θ_0	θ_1	θ_2	θ_3	θ_{41}	θ_{42}
	True	-2.197	0.811	0.200	0.250	3.476	0.889	1.034	-0.644	0.250	0.200
	Naive	-1.782<br>($2 \cdot 10^{-16}$)	0.571<br>($2 \cdot 10^{-16}$)	0.235<br>($6 \cdot 10^{-12}$)	0.274<br>($2 \cdot 10^{-16}$)	-0.716<br>($2 \cdot 10^{-16}$)	0.890<br>(0.964)	1.042<br>(0.277)	-0.641<br>(0.727)	0.255<br>(0.203)	0.196<br>(0.082)
Sc. 1	Controls†	-2.287<br>($2 \cdot 10^{-16}$)	0.840<br>($6 \cdot 10^{-4}$)	0.197<br>(0.723)	0.241<br>(0.044)	-0.716<br>($2 \cdot 10^{-16}$)	0.890<br>(0.964)	1.042<br>(0.277)	-0.641<br>(0.727)	0.255<br>(0.203)	0.196<br>(0.082)
	IPW	-2.226<br>($6 \cdot 10^{-5}$)	0.815<br>(0.595)	0.208<br>(0.267)	0.251<br>(0.882)	-3.479<br>(0.328)	0.891<br>(0.745)	1.044<br>(0.169)	-0.640<br>(0.718)	0.255<br>(0.249)	0.198<br>(0.391)
	Unified	-2.276<br>($2 \cdot 10^{-16}$)	0.837<br>(0.002)	0.193<br>(0.182)	0.242<br>(0.003)	-0.715<br>($2 \cdot 10^{-16}$)	0.889<br>(0.969)	1.041<br>(0.320)	-0.639<br>(0.601)	0.255<br>(0.265)	0.196<br>(0.062)
	True	-2.197	0.811	0.200	0.250	-3.476	0.889	1.034	1.553	0.250	0.200
	Naive	-1.894<br>($2 \cdot 10^{-16}$)	1.692<br>($2 \cdot 10^{-16}$)	0.212<br>(0.011)	0.262<br>($1 \cdot 10^{-7}$)	-1.227<br>($2 \cdot 10^{-16}$)	0.896<br>(0.191)	1.030<br>(0.623)	1.580<br>(0.018)	0.248<br>(0.607)	0.202<br>(0.386)
Sc. 2	Controls†	-2.259<br>($1 \cdot 10^{-15}$)	0.181<br>($2 \cdot 10^{-16}$)	0.157<br>($5 \cdot 10^{-6}$)	0.215<br>($3 \cdot 10^{-13}$)	-1.227<br>($2 \cdot 10^{-16}$)	0.896<br>(0.191)	1.030<br>(0.623)	1.580<br>(0.018)	0.248<br>(0.607)	0.202<br>(0.386)
	IPW	-2.219<br>($7 \cdot 10^{-4}$)	0.827<br>(0.010)	0.203<br>(0.615)	0.255<br>(0.177)	-3.481<br>(0.297)	0.896<br>(0.182)	1.031<br>(0.785)	1.589<br>(0.003)	0.241<br>(0.128)	0.201<br>(0.608)
	Unified	-2.223<br>($4 \cdot 10^{-5}$)	0.181<br>($2 \cdot 10^{-16}$)	0.117<br>($2 \cdot 10^{-16}$)	0.185<br>($2 \cdot 10^{-16}$)	-1.226<br>($2 \cdot 10^{-16}$)	0.901<br>(0.027)	1.046<br>(0.142)	1.557<br>(0.706)	0.243<br>(0.105)	0.198<br>(0.369)
	True	-2.197	0.811	0.200	0.250	-1.901	0.166	0.086	-0.793	0.250	0.200
	Naive	-2.168<br>($2 \cdot 10^{-8}$)	0.554<br>($2 \cdot 10^{-16}$)	0.187<br>(0.028)	0.246<br>(0.196)	-0.167<br>($2 \cdot 10^{-16}$)	0.169<br>(0.632)	0.084<br>(0.867)	-0.796<br>(0.844)	0.254<br>(0.374)	0.203<br>(0.219)
Sc. 3	Controls†	-2.234<br>($6 \cdot 10^{-7}$)	0.924<br>($2 \cdot 10^{-16}$)	0.200<br>(0.989)	0.260<br>(0.017)	-0.167<br>($2 \cdot 10^{-16}$)	0.169<br>(0.632)	0.084<br>(0.867)	-0.796<br>(0.844)	0.254<br>(0.374)	0.203<br>(0.219)
	IPW	-2.209<br>(0.069)	0.814<br>(0.612)	0.193<br>(0.315)	0.253<br>(0.437)	-1.904<br>(0.357)	0.169<br>(0.588)	0.086<br>(0.992)	-0.797<br>(0.760)	0.254<br>(0.367)	0.203<br>(0.091)
	Unified	-2.231<br>($8 \cdot 10^{-7}$)	0.921<br>($2 \cdot 10^{-16}$)	0.205<br>(0.399)	0.261<br>($3 \cdot 10^{-4}$)	-0.166<br>($2 \cdot 10^{-16}$)	0.167<br>(0.851)	0.081<br>(0.557)	-0.788<br>(0.660)	0.253<br>(0.518)	0.202<br>(0.383)
	True	0.201	-2.643	0.200	0.250	-1.153	0.489	-0.582	-1.068	0.250	0.200
	Naive	0.082<br>($2 \cdot 10^{-16}$)	-2.883<br>($2 \cdot 10^{-16}$)	0.196<br>(0.446)	0.240<br>($5 \cdot 10^{-5}$)	-0.116<br>($2 \cdot 10^{-16}$)	0.486<br>(0.570)	-0.588<br>(0.274)	-1.155<br>($2 \cdot 10^{-4}$)	0.253<br>(0.491)	0.199<br>(0.526)
Sc. 4	Controls†	0.314<br>($2 \cdot 10^{-16}$)	-2.440<br>($2 \cdot 10^{-16}$)	0.244<br>($1 \cdot 10^{-10}$)	0.280<br>($2 \cdot 10^{-16}$)	-0.116<br>($2 \cdot 10^{-16}$)	0.486<br>(0.570)	-0.588<br>(0.274)	-1.155<br>($2 \cdot 10^{-4}$)	0.253<br>(0.491)	0.199<br>(0.526)
	IPW	0.202<br>(0.742)	-2.667<br>(0.002)	0.210<br>(0.075)	0.252<br>(0.541)	-1.150<br>(0.472)	0.486<br>(0.513)	-0.589<br>(0.221)	-1.153<br>($2 \cdot 10^{-4}$)	0.253<br>(0.530)	0.199<br>(0.554)
	Unified	0.313<br>($2 \cdot 10^{-16}$)	-2.433<br>($2 \cdot 10^{-16}$)	0.241<br>($5 \cdot 10^{-16}$)	0.276<br>($2 \cdot 10^{-16}$)	-0.115<br>($2 \cdot 10^{-16}$)	0.485<br>(0.353)	-0.589<br>(0.166)	-1.141<br>(0.001)	0.252<br>(0.668)	0.198<br>(0.241)
	True	0.201	-2.643	0.200	0.250	-1.992	1.792	-0.450	-1.547	0.250	0.200
	Naive	0.131<br>($2 \cdot 10^{-16}$)	-3.077<br>($2 \cdot 10^{-16}$)	0.192<br>(0.145)	0.237<br>($2 \cdot 10^{-6}$)	-0.843<br>($2 \cdot 10^{-16}$)	1.793<br>(0.775)	-0.458<br>(0.244)	-1.621<br>(0.005)	0.249<br>(0.839)	0.201<br>(0.783)
Sc. 5	Controls†	0.244<br>($2 \cdot 10^{-16}$)	-2.174<br>($2 \cdot 10^{-16}$)	0.229<br>($1 \cdot 10^{-5}$)	0.270<br>($2 \cdot 10^{-9}$)	-0.843<br>($2 \cdot 10^{-16}$)	1.793<br>(0.775)	-0.458<br>(0.244)	-1.621<br>(0.005)	0.249<br>(0.839)	0.201<br>(0.783)
	IPW	0.203<br>(0.578)	-2.665<br>(0.004)	0.204<br>(0.450)	0.248<br>(0.529)	-1.990<br>(0.585)	1.794<br>(0.633)	-0.458<br>(0.243)	-1.621<br>(0.004)	0.247<br>(0.571)	0.201<br>(0.517)
	Unified	0.242<br>($2 \cdot 10^{-16}$)	-2.170<br>($2 \cdot 10^{-16}$)	0.231<br>($2 \cdot 10^{-8}$)	0.268<br>($6 \cdot 10^{-11}$)	-0.839<br>($2 \cdot 10^{-16}$)	1.790<br>(0.744)	-0.460<br>(0.121)	-1.599<br>(0.037)	0.245<br>(0.298)	0.198<br>(0.296)

† : β coefficients estimated based on the controls only and θ coefficients estimated based on all the sample (cases and controls). **Note**: p-values smaller than $2 \cdot 10^{-16}$ are shown as $2 \cdot 10^{-16}$.

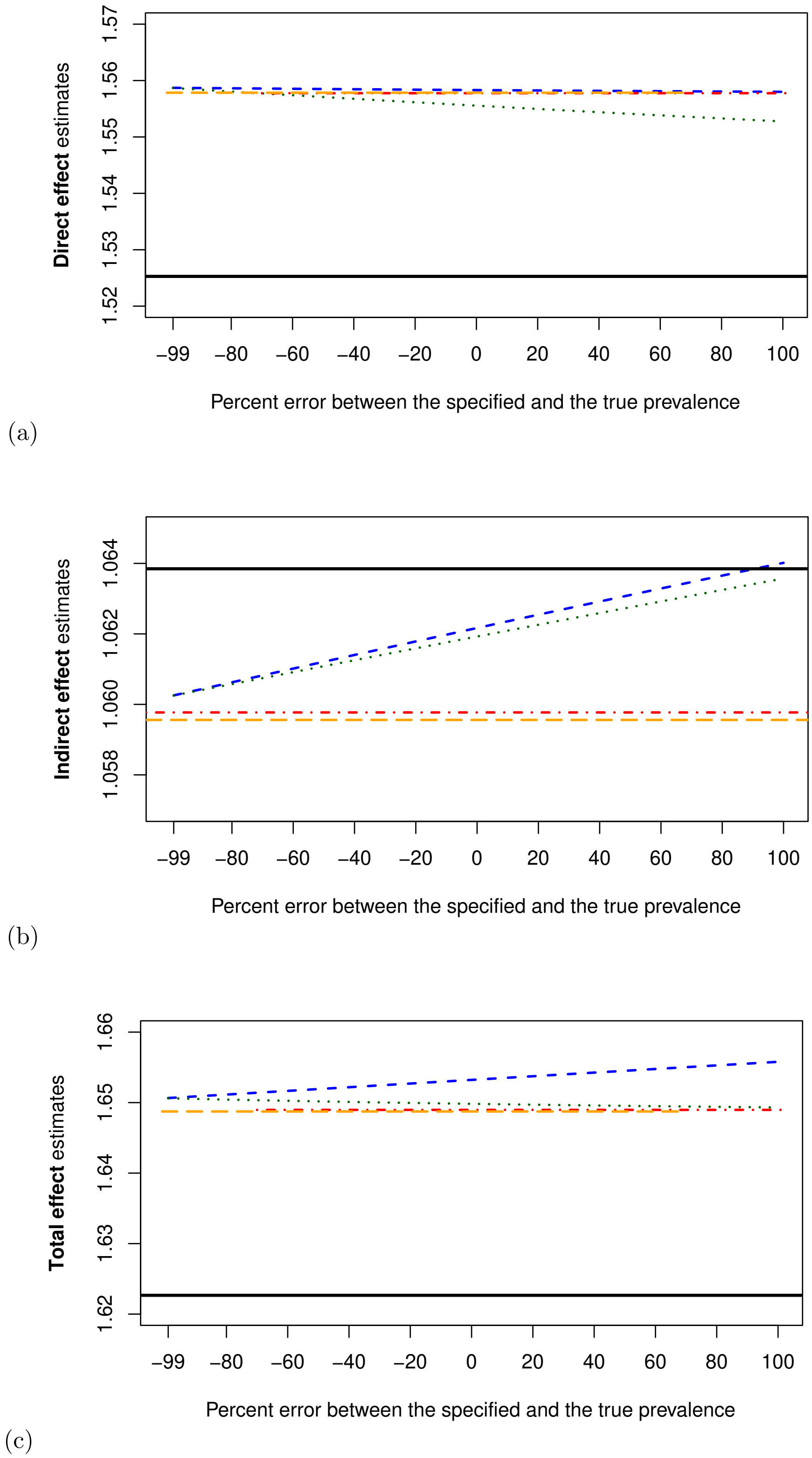

Figure A1 Scenario 1 - continuous mediator ($n = 1000$). Impact of prevalence misspecification on direct (a), indirect (b) and total (c) effects estimates. LEGEND. full line: true value; dotted line : exact approach with IPW; dashed line: approximate approach with IPW; long dashed line: unified approach; dotted dashed line: approximate approach with controls only for the mediator model.

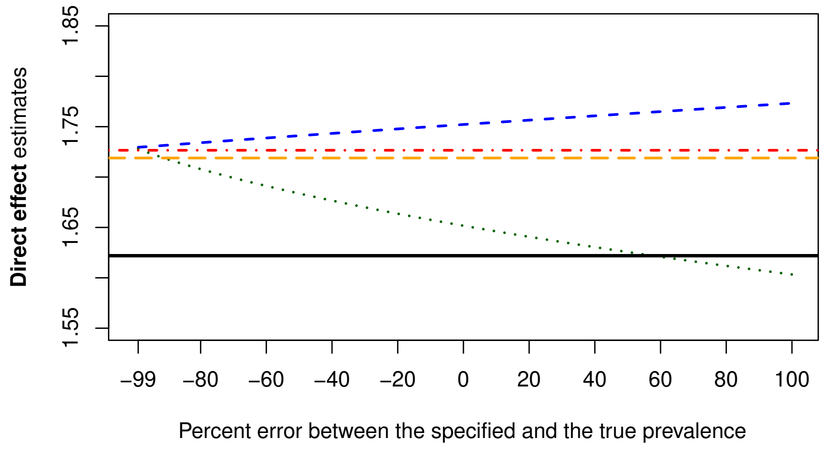

(a)

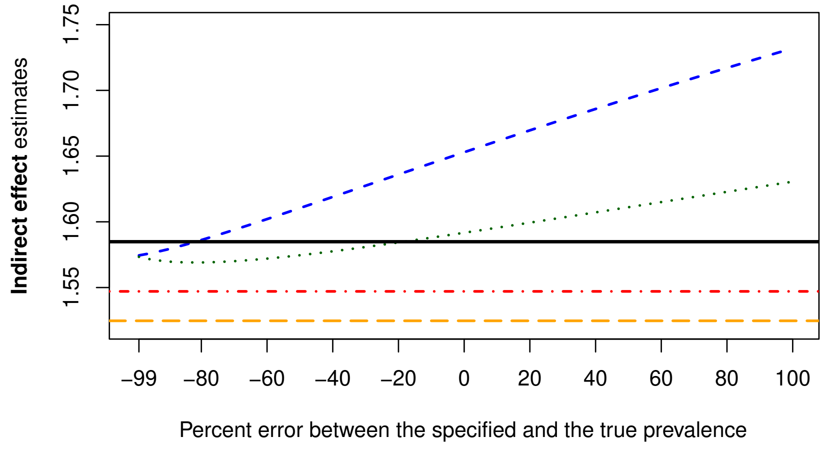

(b)

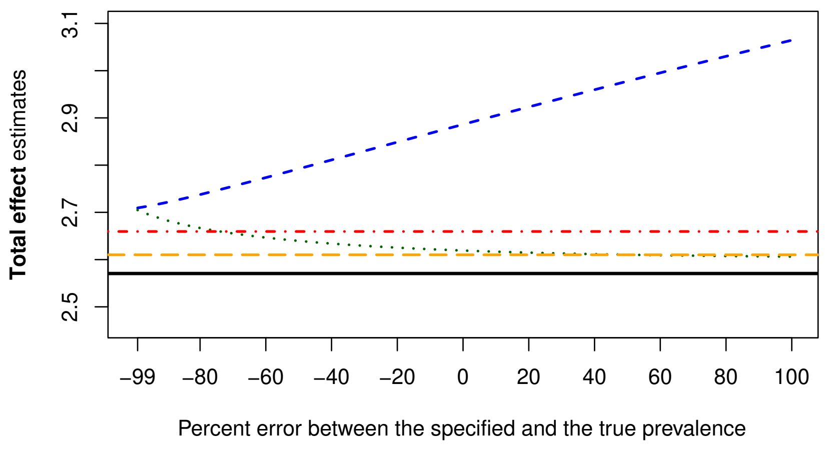

(c)

Figure A2 Scenario 2 - continuous mediator ($n = 1000$). Impact of prevalence misspecification on direct (a), indirect (b) and total (c) effects estimates. LEGEND. full line: true value; dotted line : exact approach with IPW; dashed line: approximate approach with IPW; long dashed line: unified approach; dotted dashed line: approximate approach with controls only for the mediator model.

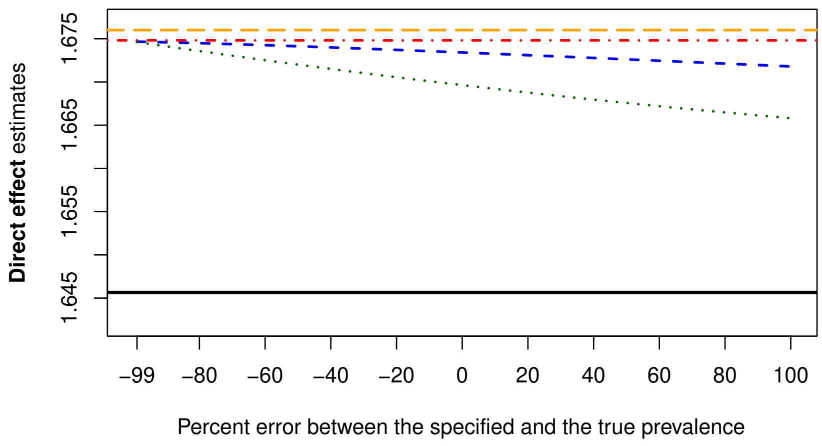

(a)

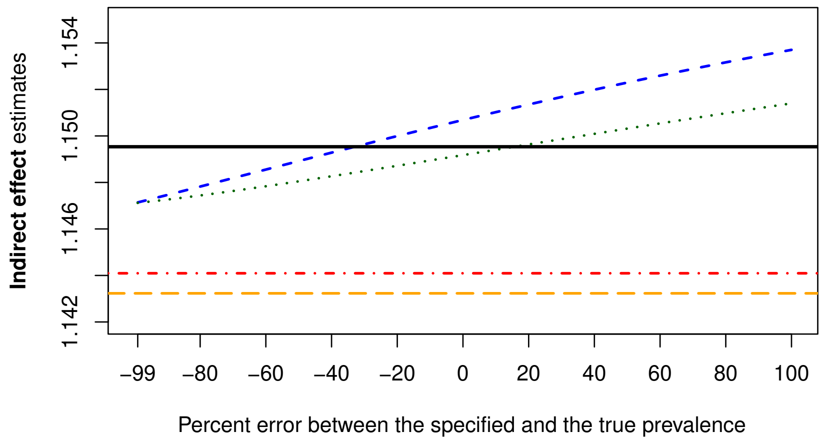

(b)

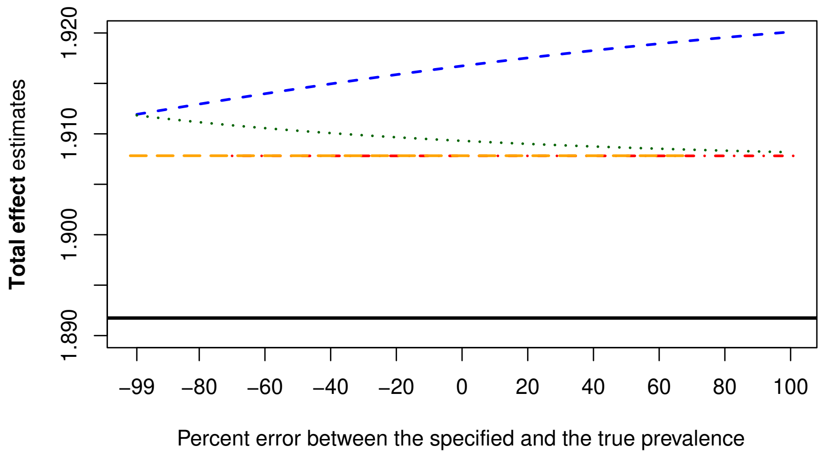

(c)

Figure A3 Scenario 3 - continuous mediator ($n = 1000$). Impact of prevalence misspecification on direct (a), indirect (b) and total (c) effects estimates. LEGEND. full line: true value; dotted line : exact approach with IPW; dashed line: approximate approach with IPW; long dashed line: unified approach; dotted dashed line: approximate approach with controls only for the mediator model.

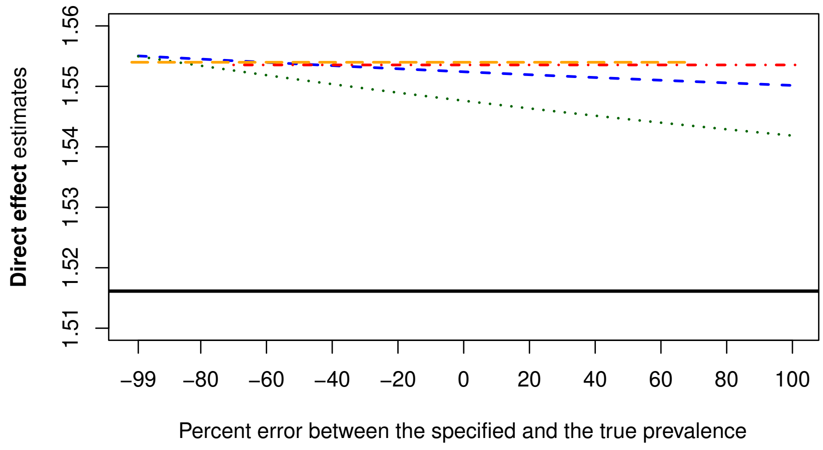

(a)

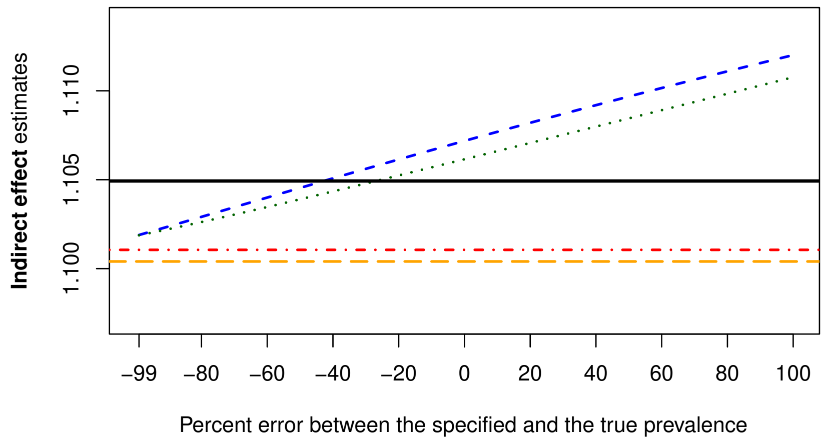

(b)

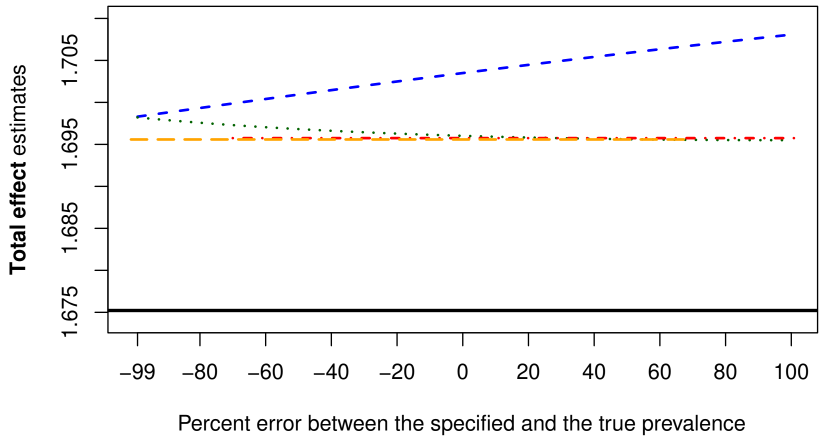

(c)

Figure A4 Scenario 4 - continuous mediator ($n = 1000$). Impact of prevalence misspecification on direct (a), indirect (b) and total (c) effects estimates. LEGEND. full line: true value; dotted line : exact approach with IPW; dashed line: approximate approach with IPW; long dashed line: unified approach; dotted dashed line: approximate approach with controls only for the mediator model.

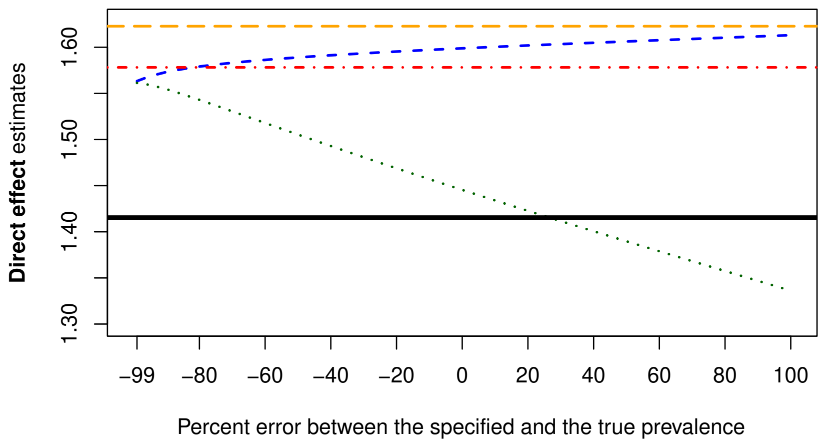

(a)

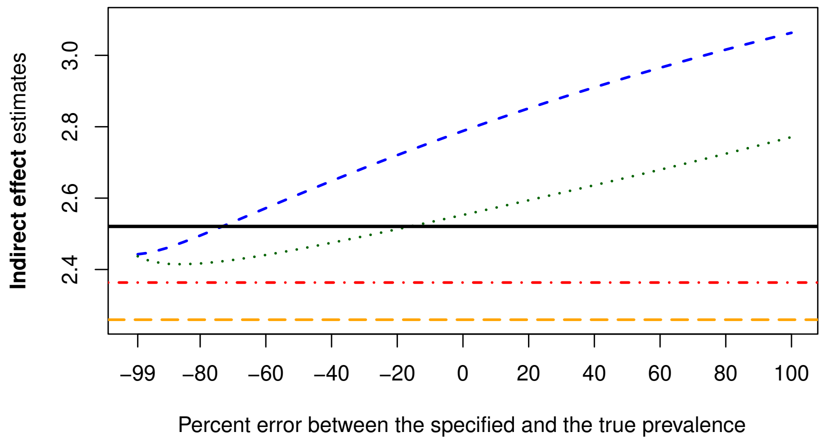

(b)

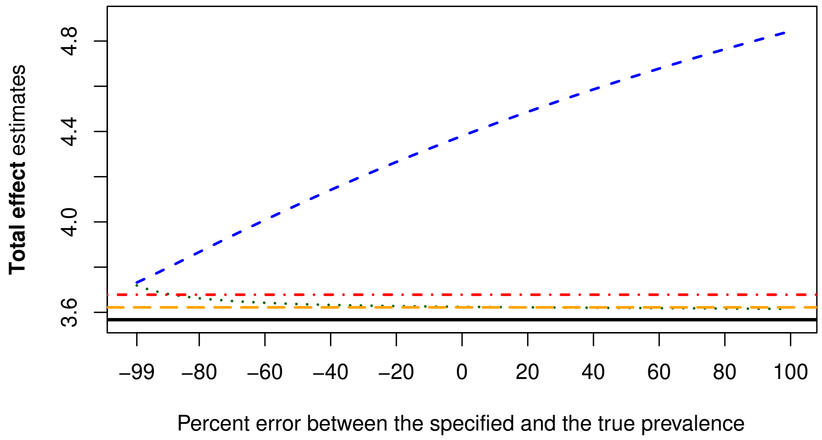

(c)

Figure A5 Scenario 5 - continuous mediator ($n = 1000$). Impact of prevalence misspecification on direct (a), indirect (b) and total (c) effects estimates. LEGEND. full line: true value; dotted line : exact approach with IPW; dashed line: approximate approach with IPW; long dashed line: unified approach; dotted dashed line: approximate approach with controls only for the mediator model.

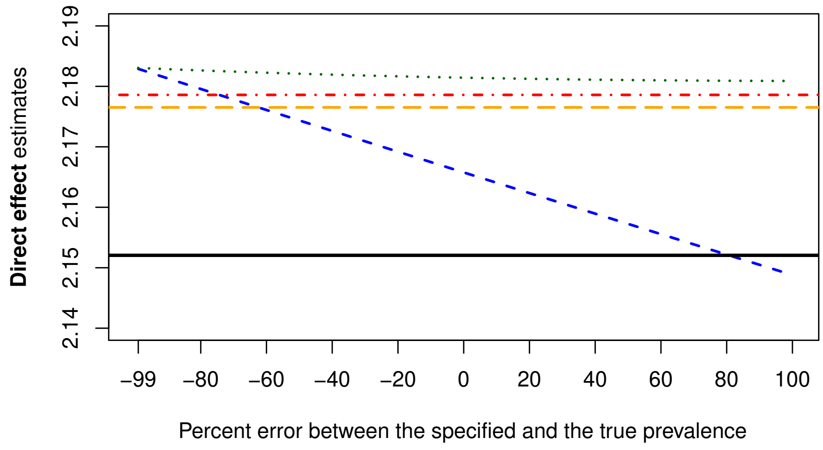

(a)

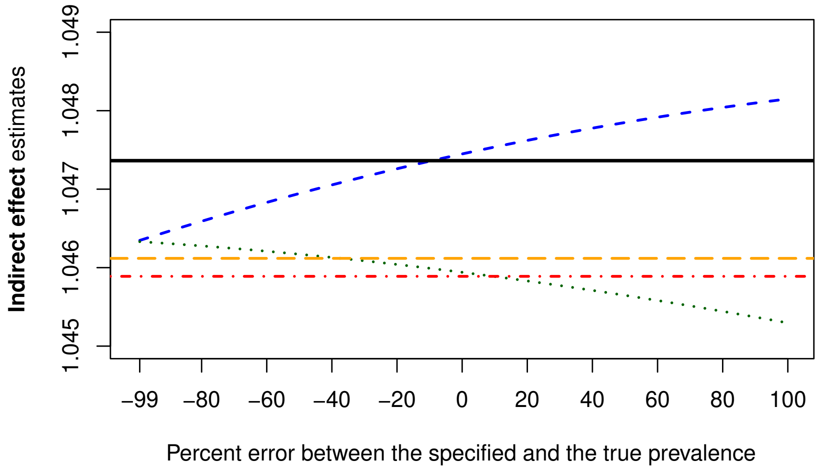

(b)

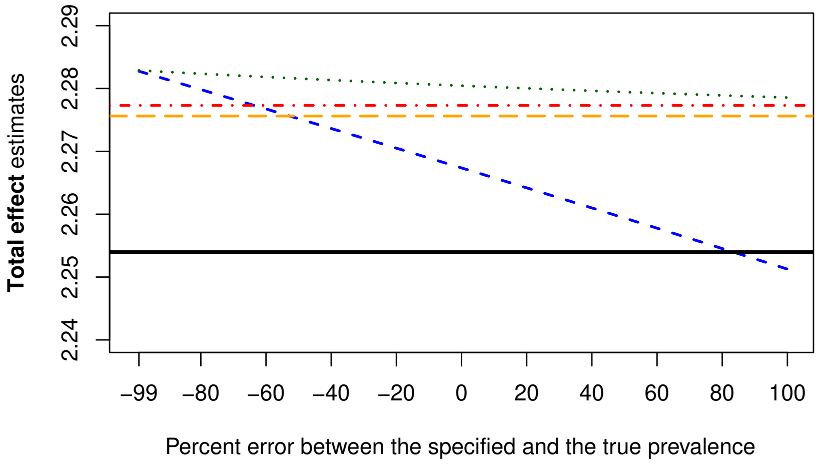

(c)

Figure A6 Scenario 1 - binary mediator ($n = 1000$). Impact of prevalence misspecification on direct (a), indirect (b) and total (c) effects estimates. LEGEND. full line: true value; dotted line : exact approach with IPW; dashed line: approximate approach with IPW; long dashed line: unified approach; dotted dashed line: approximate approach with controls only for the mediator model.

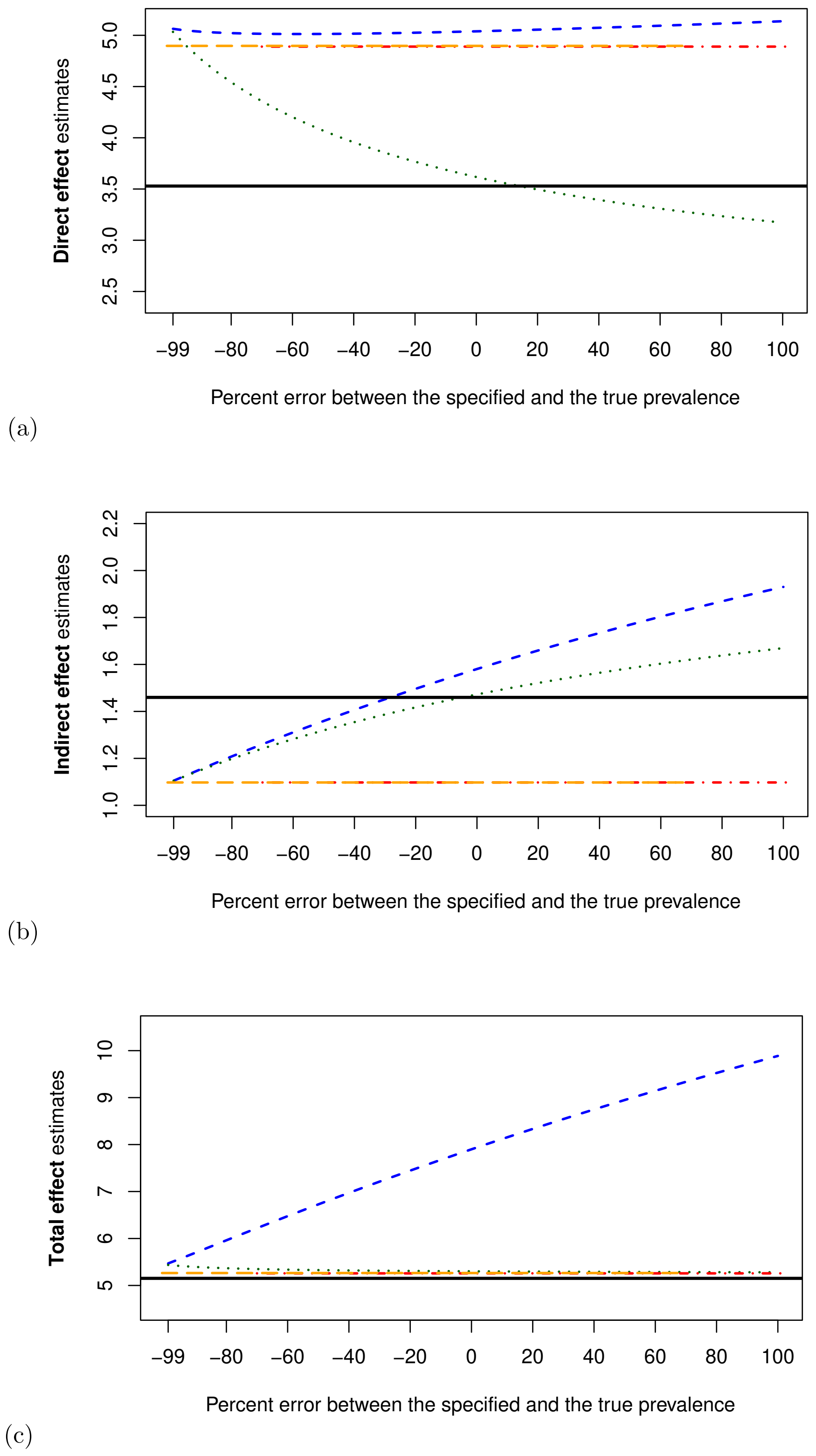

Figure A7 Scenario 2 - binary mediator ($n = 1000$). Impact of prevalence misspecification on direct (a), indirect (b) and total (c) effects estimates. LEGEND. full line: true value; dotted line : exact approach with IPW; dashed line: approximate approach with IPW; long dashed line: unified approach; dotted dashed line: approximate approach with controls only for the mediator model.

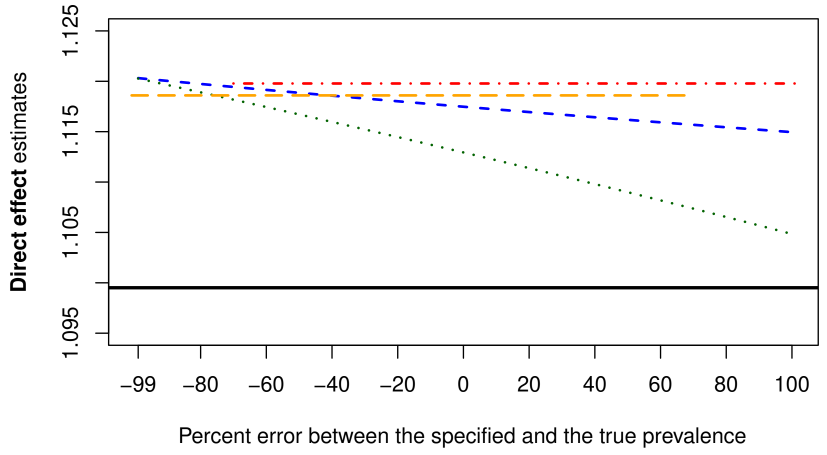

(a)

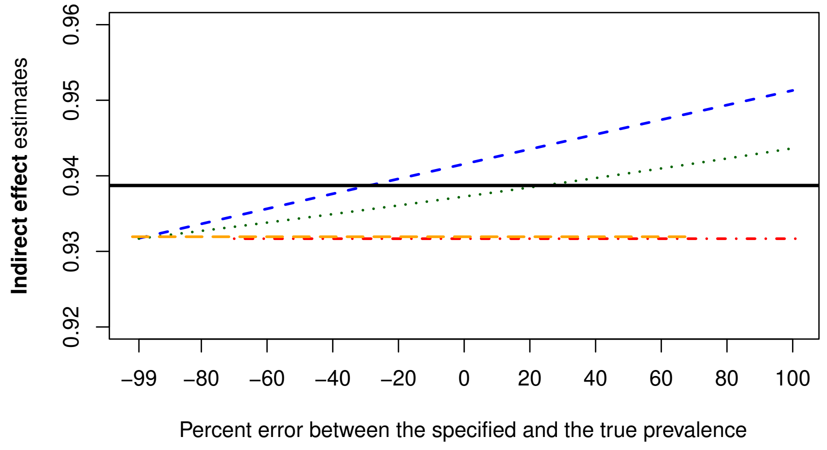

(b)

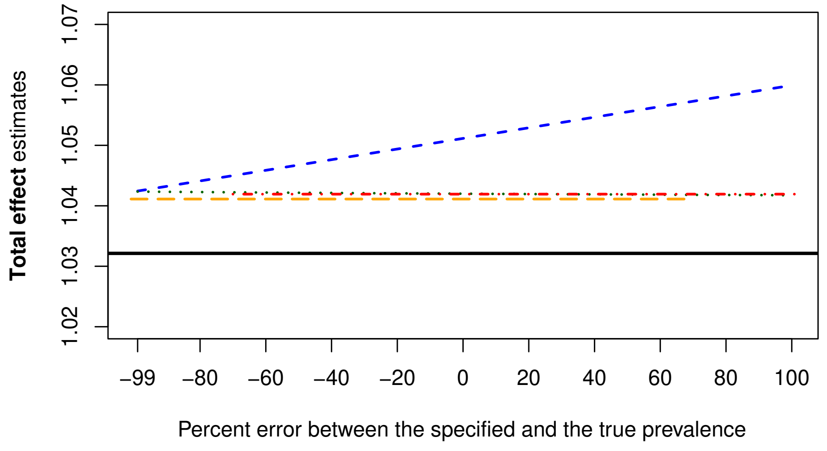

(c)

Figure A8 Scenario 3 - binary mediator ($n = 1000$). Impact of prevalence misspecification on direct (a), indirect (b) and total (c) effects estimates. LEGEND. full line: true value; dotted line : exact approach with IPW; dashed line: approximate approach with IPW; long dashed line: unified approach; dotted dashed line: approximate approach with controls only for the mediator model.

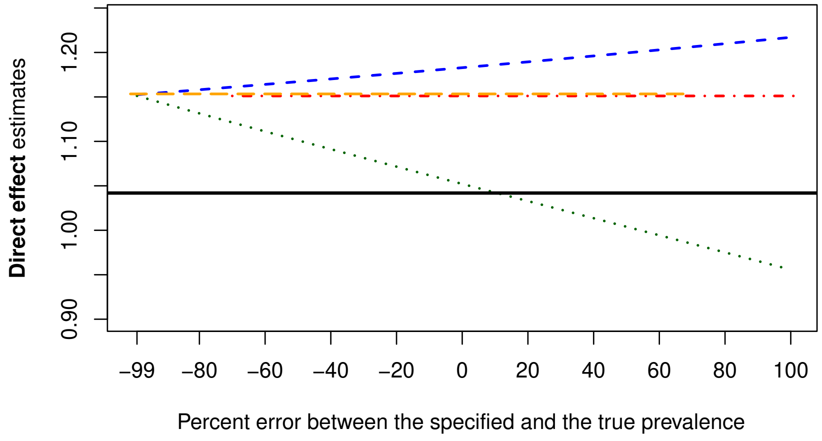

(a)

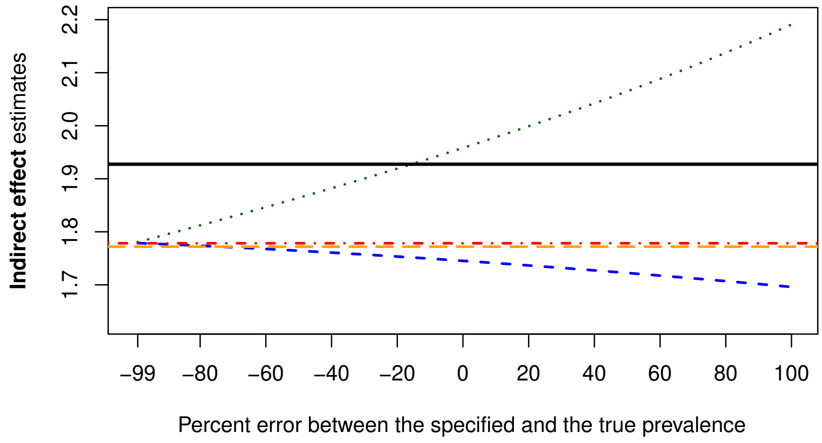

(b)

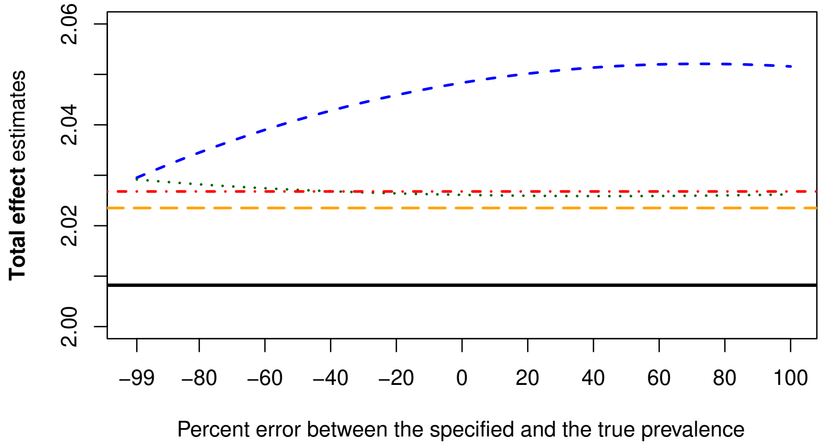

(c)

Figure A9 Scenario 4 - binary mediator ($n = 1000$). Impact of prevalence misspecification on direct (a), indirect (b) and total (c) effects estimates. LEGEND. full line: true value; dotted line : exact approach with IPW; dashed line: approximate approach with IPW; long dashed line: unified approach; dotted dashed line: approximate approach with controls only for the mediator model.

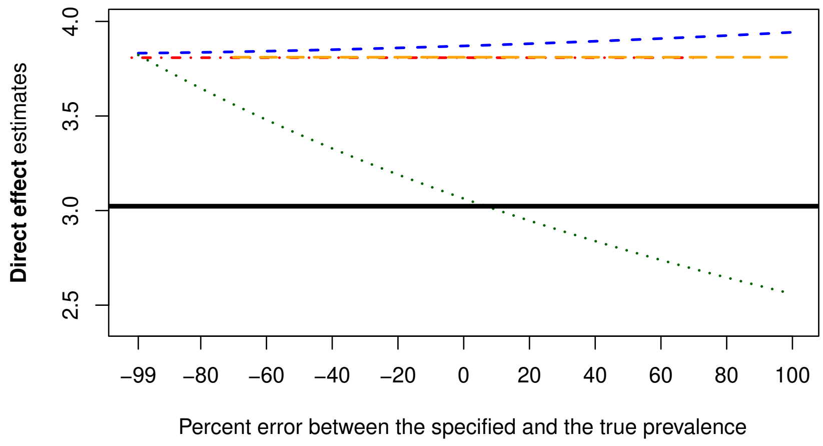

(a)

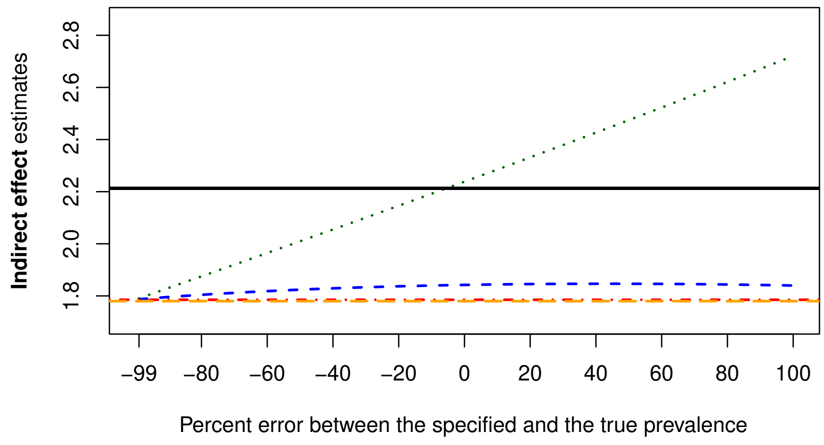

(b)

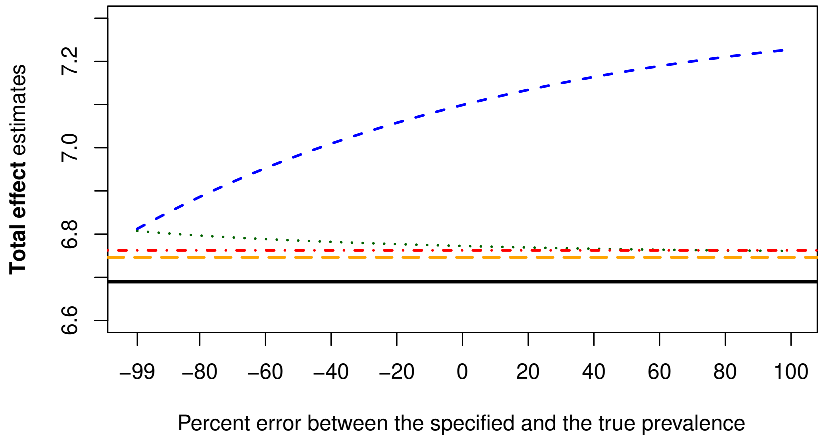

(c)

Figure A10 Scenario 5 - binary mediator ($n = 1000$). Impact of prevalence misspecification on direct (a), indirect (b) and total (c) effects estimates. LEGEND. full line: true value; dotted line : exact approach with IPW; dashed line: approximate approach with IPW; long dashed line: unified approach; dotted dashed line: approximate approach with controls only for the mediator model.
