# Supplementary material for: An empirical evaluation of approximate and exact regression-based causal mediation approaches for a binary outcome and a continuous or a binary mediator for case-control study designs
Source: BMC Med Res Methodol. 2024 Mar 20;24:72. doi: 10.1186/s12874-024-02156-y (PMC10953265; doi:10.1186/s12874-024-02156-y)
Supplement: Supplementary file 4 — Additional file 4. Simulation results with n = 500. We present the natural effects estimates obtained with the studied mediation approaches with generated samples of size n = 500. [file 12874_2024_2156_MOESM4_ESM.pdf]

Table A1 Comparison of approaches for the estimation of natural effects on the odds ratio scale for Scenario 1 with a continuous mediator (based on 1000 data sets of size $n = 500$)

Effect	Approach	True value	Mean	Bias	Relative bias (%)	SD	RMSE	CP (%) delta	CP (%) boot
NDE	Approx_Naive	1.525	1.565	0.039	2.573	0.304	0.306	95.0	94.8
	Approx_C	1.525	1.569	0.044	2.886	0.307	0.310	94.9	94.8
	Approx_IPW	1.525	1.570	0.045	2.936	0.311	0.314	95.3	95.2
	Exact_Naive	1.525	1.546	0.021	1.390	0.307	0.307	94.8	94.9
	Exact_IPW	1.525	1.567	0.041	2.710	0.311	0.313	95.8	94.5
	Unified	1.525	1.570	0.044	2.901	0.307	0.310	94.9	-
NIE	Approx_Naive	1.064	1.073	0.009	0.835	0.068	0.069	96.1	95.1
	Approx_C	1.064	1.055	-0.009	-0.825	0.053	0.054	98.4	95.4
	Approx_IPW	1.064	1.059	-0.005	-0.488	0.057	0.057	98.5	95.6
	Exact_Naive	1.064	1.071	0.007	0.687	0.066	0.066	95.5	94.7
	Exact_IPW	1.064	1.058	-0.006	-0.523	0.057	0.057	98.8	95.6
	Unified	1.064	1.054	-0.009	-0.892	0.052	0.053	98.1	-
TE	Approx_Naive	1.623	1.674	0.051	3.173	0.319	0.323	95.2	95.2
	Approx_C	1.623	1.651	0.029	1.769	0.309	0.310	95.2	94.4
	Approx_IPW	1.623	1.657	0.035	2.138	0.313	0.315	95.4	94.9
	Exact_Naive	1.623	1.650	0.028	1.702	0.308	0.309	94.9	94.2
	Exact_IPW	1.623	1.653	0.030	1.865	0.311	0.313	95.6	94.6
	Unified	1.623	1.650	0.028	1.715	0.308	0.309	94.9	-

boot: bootstrap; CP: coverage probability; NDE: natural direct effect; NIE: natural indirect effect; RMSE: root mean squared error; SD: standard deviation; TE: total effect.

Table A2 Comparison of approaches for the estimation of natural effects on the odds ratio scale for Scenario 2 with a continuous mediator (based on 1000 data sets of size $n = 500$)

Effect	Approach	True value	Mean	Bias	Relative bias (%)	SD	RMSE	CP (%) delta	CP (%) boot
NDE	Approx_Naive	1.622	1.872	0.250	15.422	0.418	0.488	91.3	90.6
	Approx_C	1.622	1.750	0.129	7.923	0.378	0.399	92.3	92.1
	Approx_IPW	1.622	1.780	0.158	9.754	0.395	0.426	92.4	92.2
	Exact_Naive	1.622	1.507	-0.115	-7.111	0.297	0.318	91.8	91.7
	Exact_IPW	1.622	1.665	0.043	2.667	0.346	0.349	94.3	94.1
	Unified	1.622	1.739	0.117	7.232	0.371	0.389	93.6	-
NIE	Approx_Naive	1.585	1.939	0.354	22.362	0.283	0.453	81.1	70.9
	Approx_C	1.585	1.556	-0.029	-1.822	0.164	0.167	97.9	92.6
	Approx_IPW	1.585	1.678	0.093	5.848	0.204	0.224	98.7	92.5
	Exact_Naive	1.585	1.754	0.169	10.695	0.180	0.247	87.5	84.7
	Exact_IPW	1.585	1.608	0.023	1.446	0.168	0.170	98.7	94.4
	Unified	1.585	1.528	-0.057	-3.603	0.142	0.153	91.3	-
TE	Approx_Naive	2.571	3.641	1.070	41.642	1.032	1.487	84.6	79.3
	Approx_C	2.571	2.697	0.127	4.935	0.529	0.544	98.3	94.5
	Approx_IPW	2.571	2.964	0.393	15.291	0.656	0.764	96.5	89.8
	Exact_Naive	2.571	2.626	0.055	2.155	0.502	0.505	94.8	94.8
	Exact_IPW	2.571	2.655	0.085	3.299	0.515	0.522	97.4	94.5
	Unified	2.571	2.634	0.064	2.474	0.504	0.508	94.8	-

boot: bootstrap; CP: coverage probability; NDE: natural direct effect; NIE: natural indirect effect; RMSE: root mean squared error; SD: standard deviation; TE: total effect.

Table A3 Comparison of approaches for the estimation of natural effects on the odds ratio scale for Scenario 3 with a continuous mediator (based on 1000 data sets of size $n = 500$)

Effect	Approach	True value	Mean	Bias	Relative bias (%)	SD	RMSE	CP (%) delta	CP (%) boot
NDE	Approx_Naive	1.646	1.701	0.055	3.333	0.355	0.359	95.8	95.2
	Approx_C	1.646	1.711	0.066	3.996	0.362	0.368	95.6	95.5
	Approx_IPW	1.646	1.711	0.065	3.971	0.363	0.369	95.3	94.6
	Exact_Naive	1.646	1.693	0.047	2.866	0.362	0.365	95.4	95.1
	Exact_IPW	1.646	1.706	0.061	3.696	0.365	0.370	95.6	94.9
	Unified	1.646	1.712	0.067	4.058	0.362	0.368	95.7	-
NIE	Approx_Naive	1.150	1.161	0.012	1.005	0.132	0.132	96.4	96.0
	Approx_C	1.150	1.143	-0.006	-0.558	0.110	0.110	98.4	95.6
	Approx_IPW	1.150	1.152	0.002	0.175	0.121	0.121	98.0	95.8
	Exact_Naive	1.150	1.157	0.008	0.670	0.126	0.126	96.0	95.9
	Exact_IPW	1.150	1.149	0.000	-0.020	0.117	0.117	98.2	96.2
	Unified	1.150	1.142	-0.008	-0.672	0.106	0.107	96.3	-
TE	Approx_Naive	1.892	1.959	0.067	3.564	0.392	0.398	94.6	94.1
	Approx_C	1.892	1.940	0.049	2.566	0.371	0.374	94.9	94.3
	Approx_IPW	1.892	1.954	0.062	3.270	0.381	0.386	95.4	94.6
	Exact_Naive	1.892	1.939	0.048	2.521	0.370	0.373	94.2	94.2
	Exact_IPW	1.892	1.943	0.052	2.734	0.372	0.376	95.3	94.4
	Unified	1.892	1.940	0.048	2.536	0.370	0.373	94.9	-

boot: bootstrap; CP: coverage probability; NDE: natural direct effect; NIE: natural indirect effect; RMSE: root mean squared error; SD: standard deviation; TE: total effect.

Table A4 Comparison of approaches for the estimation of natural effects on the odds ratio scale for Scenario 4 with a continuous mediator (based on 1000 data sets of size $n = 500$)

Effect	Approach	True value	Mean	Bias	Relative bias (%)	SD	RMSE	CP (%) delta	CP (%) boot
NDE	Approx_Naive	1.516	1.582	0.066	4.358	0.367	0.372	95.5	95.4
	Approx_C	1.516	1.590	0.074	4.860	0.370	0.377	95.7	95.4
	Approx_IPW	1.516	1.587	0.071	4.698	0.370	0.377	95.5	95.3
	Exact_Naive	1.516	1.573	0.056	3.718	0.368	0.373	95.5	95.0
	Exact_IPW	1.516	1.581	0.065	4.273	0.370	0.375	95.7	95.1
	Unified	1.516	1.589	0.073	4.829	0.367	0.374	95.6	-
NIE	Approx_Naive	1.105	1.114	0.009	0.787	0.159	0.159	95.6	94.6
	Approx_C	1.105	1.097	-0.007	-0.676	0.139	0.139	96.8	95.0
	Approx_IPW	1.105	1.107	0.002	0.213	0.151	0.151	96.1	94.3
	Exact_Naive	1.105	1.111	0.006	0.585	0.154	0.154	94.8	94.7
	Exact_IPW	1.105	1.105	0.001	0.050	0.148	0.148	95.8	94.7
	Unified	1.105	1.097	-0.008	-0.718	0.137	0.137	95.6	-
TE	Approx_Naive	1.675	1.731	0.056	3.341	0.331	0.336	95.4	94.8
	Approx_C	1.675	1.716	0.040	2.411	0.321	0.324	95.9	95.0
	Approx_IPW	1.675	1.727	0.052	3.088	0.327	0.331	95.6	94.6
	Exact_Naive	1.675	1.715	0.040	2.376	0.320	0.323	95.6	95.0
	Exact_IPW	1.675	1.716	0.041	2.453	0.321	0.323	96.0	95.8
	Unified	1.675	1.715	0.040	2.382	0.320	0.323	95.6	-

boot: bootstrap; CP: coverage probability; NDE: natural direct effect; NIE: natural indirect effect; RMSE: root mean squared error; SD: standard deviation; TE: total effect.

Table A5 Comparison of approaches for the estimation of natural effects on the odds ratio scale for Scenario 5 with a continuous mediator (based on 1000 data sets of size $n = 500$)

Effect	Approach	True value	Mean	Bias	Relative bias (%)	SD	RMSE	CP (%) delta	CP (%) boot
NDE	Approx_Naive	1.415	1.632	0.217	15.321	0.404	0.459	94.5	93.7
	Approx_C	1.415	1.606	0.190	13.448	0.414	0.455	95.3	94.5
	Approx_IPW	1.415	1.624	0.209	14.742	0.413	0.462	94.6	93.7
	Exact_Naive	1.415	1.390	-0.025	-1.800	0.400	0.401	94.6	95.0
	Exact_IPW	1.415	1.469	0.054	3.824	0.407	0.410	95.5	95.0
	Unified	1.415	1.648	0.233	16.471	0.420	0.480	94.5	-
NIE	Approx_Naive	2.521	3.088	0.567	22.503	0.897	1.061	93.7	90.2
	Approx_C	2.521	2.394	-0.127	-5.020	0.438	0.456	98.3	94.6
	Approx_IPW	2.521	2.865	0.344	13.643	0.745	0.820	96.8	92.2
	Exact_Naive	2.521	2.745	0.224	8.903	0.586	0.627	93.9	93.0
	Exact_IPW	2.521	2.588	0.068	2.684	0.511	0.515	97.1	95.7
	Unified	2.521	2.278	-0.243	-9.634	0.365	0.439	91.4	-
TE	Approx_Naive	3.568	4.853	1.285	36.023	1.235	1.783	83.2	76.3
	Approx_C	3.568	3.734	0.167	4.668	0.736	0.755	97.9	95.0
	Approx_IPW	3.568	4.486	0.918	25.731	1.054	1.398	89.6	83.2
	Exact_Naive	3.568	3.654	0.086	2.417	0.702	0.708	95.4	95.0
	Exact_IPW	3.568	3.665	0.097	2.723	0.707	0.714	95.7	94.8
	Unified	3.568	3.660	0.092	2.588	0.705	0.711	95.4	-

boot: bootstrap; CP: coverage probability; NDE: natural direct effect; NIE: natural indirect effect; RMSE: root mean squared error; SD: standard deviation; TE: total effect.

Table A6 Comparison of approaches for the estimation of natural effects on the odds ratio scale for Scenario 1 with a binary mediator (based on 1000 data sets of size $n = 500$)

Effect	Approach	True value	Mean	Bias	Relative bias (%)	SD	RMSE	CP (%) delta	CP (%) boot
NDE	Approx_Naive	2.152	2.065	-0.087	-4.044	0.432	0.440	94.3	93.3
	Approx_C	2.152	2.220	0.068	3.178	0.432	0.437	95.5	94.1
	Approx_IPW	2.152	2.208	0.056	2.613	0.443	0.446	95.0	94.7
	Exact_Naive	2.152	2.223	0.071	3.313	0.425	0.431	94.9	94.8
	Exact_IPW	2.152	2.224	0.072	3.363	0.442	0.448	95.6	94.4
	Unified	2.152	2.216	0.064	2.971	0.431	0.435	94.7	-
NIE	Approx_Naive	1.047	1.045	-0.002	-0.201	0.044	0.044	88.6	93.6
	Approx_C	1.047	1.041	-0.006	-0.620	0.036	0.037	97.3	95.6
	Approx_IPW	1.047	1.044	-0.004	-0.358	0.038	0.038	97.3	95.9
	Exact_Naive	1.047	1.036	-0.011	-1.068	0.033	0.035	87.6	91.0
	Exact_IPW	1.047	1.042	-0.005	-0.519	0.036	0.037	97.4	96.0
	Unified	1.047	1.041	-0.006	-0.581	0.036	0.036	97.9	-
TE	Approx_Naive	2.254	2.160	-0.094	-4.176	0.467	0.476	93.5	92.9
	Approx_C	2.254	2.308	0.054	2.411	0.440	0.443	95.1	95.0
	Approx_IPW	2.254	2.302	0.048	2.132	0.455	0.458	95.2	94.2
	Exact_Naive	2.254	2.303	0.049	2.158	0.439	0.441	94.2	94.1
	Exact_IPW	2.254	2.315	0.061	2.710	0.453	0.457	95.1	93.8
	Unified	2.254	2.305	0.051	2.247	0.439	0.442	94.2	-

boot: bootstrap; CP: coverage probability; NDE: natural direct effect; NIE: natural indirect effect; RMSE: root mean squared error; SD: standard deviation; TE: total effect.

Table A7 Comparison of approaches for the estimation of natural effects on the odds ratio scale for Scenario 2 with a binary mediator (based on 1000 data sets of size $n = 500$)

Effect	Approach	True value	Mean	Bias	Relative bias (%)	SD	RMSE	CP (%) delta	CP (%) boot
NDE	Approx_Naive	3.529	5.966	2.437	69.048	2.330	3.372	70.8	63.3
	Approx_C	3.529	5.172	1.644	46.574	1.961	2.558	81.6	76.7
	Approx_IPW	3.529	5.381	1.852	52.486	2.057	2.768	78.1	71.7
	Exact_Naive	3.529	2.760	-0.769	-21.779	0.618	0.986	72.2	75.4
	Exact_IPW	3.529	3.691	0.162	4.595	0.859	0.874	93.8	93.7
	Unified	3.529	5.195	1.666	47.214	1.964	2.575	83.1	-
NIE	Approx_Naive	1.460	2.502	1.402	71.415	0.378	1.109	1.4	1.0
	Approx_C	1.460	1.087	-0.373	-25.564	0.238	0.442	77.5	60.4
	Approx_IPW	1.460	1.587	0.127	8.720	0.240	0.272	97.5	90.4
	Exact_Naive	1.460	1.937	0.477	32.683	0.201	0.518	18.4	13.5
	Exact_IPW	1.460	1.474	0.014	0.968	0.182	0.183	98.6	93.7
	Unified	1.460	1.087	-0.373	-25.535	0.236	0.441	82.0	-
TE	Approx_Naive	5.151	15.061	9.910	192.391	6.879	12.063	16.0	12.5
	Approx_C	5.151	5.292	0.141	2.739	1.127	1.136	99.6	94.8
	Approx_IPW	5.151	8.337	3.186	61.845	2.637	4.135	82.1	57.0
	Exact_Naive	5.151	5.305	0.154	2.984	1.122	1.132	93.7	93.6
	Exact_IPW	5.151	5.379	0.228	4.420	1.163	1.185	97.3	94.1
	Unified	5.151	5.320	0.169	3.273	1.127	1.139	93.8	-

boot: bootstrap; CP: coverage probability; NDE: natural direct effect; NIE: natural indirect effect; RMSE: root mean squared error; SD: standard deviation; TE: total effect.

Table A8 Comparison of approaches for the estimation of natural effects on the odds ratio scale for Scenario 3 with a binary mediator (based on 1000 data sets of size $n = 500$)

Effect	Approach	True value	Mean	Bias	Relative bias (%)	SD	RMSE	CP (%) delta	CP (%) boot
NDE	Approx_Naive	1.100	1.132	0.033	2.982	0.226	0.229	94.9	94.6
	Approx_C	1.100	1.144	0.044	4.005	0.228	0.233	95.2	94.5
	Approx_IPW	1.100	1.141	0.042	3.800	0.228	0.232	95.4	95.1
	Exact_Naive	1.100	1.112	0.013	1.163	0.214	0.214	95.4	94.3
	Exact_IPW	1.100	1.137	0.037	3.403	0.225	0.228	95.9	94.7
	Unified	1.100	1.142	0.042	3.851	0.228	0.231	95.1	-
NIE	Approx_Naive	0.939	0.965	0.026	2.757	0.023	0.035	73.5	78.7
	Approx_C	0.939	0.929	-0.009	-0.993	0.046	0.047	87.0	96.8
	Approx_IPW	0.939	0.940	0.001	0.150	0.038	0.038	86.7	94.8
	Exact_Naive	0.939	0.953	0.014	1.526	0.033	0.036	85.9	91.0
	Exact_IPW	0.939	0.935	-0.004	-0.384	0.043	0.043	88.5	95.2
	Unified	0.939	0.930	-0.009	-0.960	0.046	0.047	92.3	-
TE	Approx_Naive	1.032	1.091	0.059	5.709	0.213	0.221	94.7	94.3
	Approx_C	1.032	1.059	0.027	2.633	0.199	0.201	96.1	95.4
	Approx_IPW	1.032	1.070	0.038	3.700	0.204	0.207	96.2	95.2
	Exact_Naive	1.032	1.058	0.026	2.554	0.198	0.200	95.1	94.2
	Exact_IPW	1.032	1.060	0.028	2.723	0.199	0.201	96.3	95.4
	Unified	1.032	1.058	0.026	2.513	0.198	0.200	95.0	-

boot: bootstrap; CP: coverage probability; NDE: natural direct effect; NIE: natural indirect effect; RMSE: root mean squared error; SD: standard deviation; TE: total effect.

Table A9 Comparison of approaches for the estimation of natural effects on the odds ratio scale for Scenario 4 with a binary mediator (based on 1000 data sets of size $n = 500$)

Effect	Approach	True value	Mean	Bias	Relative bias (%)	SE	RMSE	CP (%) delta	CP (%) boot
NDE	Approx_Naive	1.042	1.256	0.214	20.567	0.334	0.396	91.1	89.1
	Approx_C	1.042	1.200	0.158	15.149	0.351	0.384	93.7	93.3
	Approx_IPW	1.042	1.230	0.188	18.045	0.343	0.391	92.4	91.7
	Exact_Naive	1.042	1.005	-0.037	-3.553	0.346	0.348	90.0	91.3
	Exact_IPW	1.042	1.097	0.055	5.264	0.336	0.340	94.8	94.4
	Unified	1.042	1.203	0.161	15.440	0.358	0.393	94.1	-
NIE	Approx_Naive	1.927	1.691	-0.237	-12.287	0.213	0.319	91.4	84.6
	Approx_C	1.927	1.768	-0.160	-8.293	0.252	0.299	96.5	92.5
	Approx_IPW	1.927	1.734	-0.194	-10.051	0.230	0.301	95.5	90.5
	Exact_Naive	1.927	2.185	0.258	13.370	0.547	0.605	89.0	90.8
	Exact_IPW	1.927	1.956	0.028	1.460	0.361	0.362	95.7	94.4
	Unified	1.927	1.761	-0.167	-8.658	0.250	0.300	93.6	-
TE	Approx_Naive	2.008	2.089	0.081	4.033	0.425	0.433	94.3	93.6
	Approx_C	2.008	2.068	0.060	2.994	0.401	0.405	95.2	94.0
	Approx_IPW	2.008	2.090	0.082	4.061	0.417	0.425	95.1	93.4
	Exact_Naive	2.008	2.067	0.059	2.931	0.400	0.405	94.3	93.4
	Exact_IPW	2.008	2.069	0.061	3.027	0.401	0.406	95.1	93.1
	Unified	2.008	2.064	0.056	2.787	0.400	0.404	94.3	-

boot: bootstrap; CP: coverage probability; NDE: natural direct effect; NIE: natural indirect effect; RMSE: root mean squared error; SD: standard deviation; TE: total effect.

Table A10 Comparison of approaches for the estimation of natural effects on the odds ratio scale for Scenario 5 with a binary mediator (based on 1000 data sets of size $n = 500$)

Effects	Approach	True value	Mean	Bias	Relative bias (%)	SD	RMSE	CP (%) delta	CP (%) boot
NDE	Approx_Naive	3.023	4.128	1.105	36.552	0.961	1.464	76.9	72.6
	Approx_C	3.023	4.000	0.977	32.317	0.968	1.375	79.8	77.4
	Approx_IPW	3.023	4.067	1.044	34.519	0.971	1.425	78.6	74.7
	Exact_Naive	3.023	2.655	-0.368	-12.173	0.927	0.998	86.6	89.6
	Exact_IPW	3.023	3.219	0.196	6.475	0.882	0.904	95.3	94.0
	Unified	3.023	4.000	0.977	32.313	0.964	1.372	80.4	-
NIE	Approx_Naive	2.213	1.834	-0.378	-17.103	0.207	0.431	57.2	52.5
	Approx_C	2.213	1.779	-0.434	-19.591	0.205	0.480	56.4	47.6
	Approx_IPW	2.213	1.838	-0.375	-16.942	0.213	0.431	61.6	56.5
	Exact_Naive	2.213	2.848	0.635	28.703	0.826	1.042	81.7	83.2
	Exact_IPW	2.213	2.258	0.045	2.025	0.419	0.421	95.3	93.3
	Unified	2.213	1.775	-0.438	-19.806	0.205	0.484	44.0	-
TE	Approx_Naive	6.690	7.500	0.810	12.115	1.663	1.850	93.8	92.3
	Approx_C	6.690	7.019	0.329	4.922	1.484	1.520	95.7	94.3
	Approx_IPW	6.690	7.387	0.698	10.431	1.628	1.772	94.5	92.6
	Exact_Naive	6.690	7.016	0.327	4.884	1.480	1.516	95.1	94.5
	Exact_IPW	6.690	7.045	0.356	5.312	1.502	1.543	95.2	93.9
	Unified	6.690	7.000	0.310	4.632	1.470	1.502	95.0	-

boot: bootstrap; CP: coverage probability; NDE: natural direct effect; NIE: natural indirect effect; RMSE: root mean squared error; SD: standard deviation; TE: total effect.
