# Supplementary material for: An empirical evaluation of approximate and exact regression-based causal mediation approaches for a binary outcome and a continuous or a binary mediator for case-control study designs
Source: BMC Med Res Methodol. 2024 Mar 20;24:72. doi: 10.1186/s12874-024-02156-y (PMC10953265; doi:10.1186/s12874-024-02156-y)
Supplement: Supplementary file 5 — Additional file 5. Additional results for the real-data analysis. We present mediation analysis results based on the data from the PROVAQ study, including the estimated mediator and outcome models for the main analysis as well as results corresponding to the simpler outcome model which omits the interaction term between the exposure and mediator. [file 12874_2024_2156_MOESM5_ESM.pdf]

The estimated regression coefficients for the main mediation analysis based on data from the PROVAQ study are shown in Tables A1 and A2. This analysis considers an interaction term between the exposure (long-term use of oral contraceptives) and the mediator (lifetime number of ovulatory cycles) in the outcome model.

Table A1 Estimated regression coefficients for the lifetime number of ovulatory cycles (mediator) model using the ExactMed R package

Variables in model	Estimate	SE	P-value
Intercept	124.101	20.390	< 0.001
Long-term use of oral contraceptives (≥ 10 years)	-116.524	6.859	< 0.001
Age (years)	4.232	0.306	< 0.001
Education level ($>$ high school)	21.451	7.542	0.005

SE: standard error.

Table A2 Estimated regression coefficients for the invasive ovarian cancer (outcome) model using the ExactMed R package

Variables in model	Estimate	SE	P-value
Intercept	-9.046	0.388	< 0.001
Long-term use of oral contraceptives (≥ 10 years)	-0.123	0.457	0.79
Lifetime number of ovulatory cycles (LOC)	0.002	0.001	0.01
Long-term use of oral contraceptives $\times$ LOC	-0.001	0.001	0.59
Age (years)	-0.006	0.006	0.38
Education level ($>$ high school)	-0.254	0.139	0.07

SE: standard error.

In Tables A3-A4, we present additional mediation analysis results corresponding to the simpler outcome model which omits the interaction term between the exposure (long-term use of oral contraceptives) and mediator (lifetime number of ovulatory cycles).

Table A3 Estimated regression coefficients for the invasive ovarian cancer (outcome) model using the ExactMed R package (model without exposure-mediator interaction term)

Variables in model	Estimate	SE	P-value
Intercept	-8.998	0.364	$<$ 0.001
Long-term use of oral contraceptives (≥ 10 years)	-0.346	0.178	0.05
Lifetime number of ovulatory cycles	0.002	0.001	0.01
Age (years)	-0.006	0.006	0.37
Education level ($>$ high school)	-0.252	0.138	0.07

SE: standard error.

Table A4 Estimated conditional total effect (TE) and natural direct effect (NDE) of long-term use of oral contraceptives on invasive ovarian cancer, with natural indirect effect (NIE) via lifetime number of ovulatory cycles (outcome model without exposure-mediator interaction term)

Effects	Approach	Estimate	SE	95% CI
NDE	Approx_Naive	0.711	0.129	0.498, 1.016
	Approx_C	0.711	0.129	0.498, 1.016
	Approx_IPW	0.708	0.126	0.499, 1.003
	Exact_Naive	0.712	0.129	0.499, 1.017
	Exact_IPW	0.708	0.126	0.499, 1.003
	Unified	0.708	0.127	0.499, 1.006
NIE	Approx_Naive	0.809	0.068	0.686, 0.954
	Approx_C	0.818	0.065	0.699, 0.957
	Approx_IPW	0.814	0.067	0.693, 0.955
	Exact_Naive	0.810	0.067	0.688, 0.953
	Exact_IPW	0.814	0.067	0.693, 0.955
	Unified	0.805	0.062	0.693, 0.937
TE	Approx_Naive	0.575	0.093	0.418, 0.791
	Approx_C	0.582	0.094	0.423, 0.800
	Approx_IPW	0.576	0.094	0.419, 0.792
	Exact_Naive	0.577	0.093	0.420, 0.792
	Exact_IPW	0.576	0.094	0.418, 0.792
	Unified	0.571	0.092	0.415, 0.784

CI: confidence interval; SE: standard error.
